# An expanded palette of improved SPLICS reporters detects multiple organelle contacts in vitro and in vivo

**DOI:** 10.1038/s41467-020-19892-6

**Published:** 2020-11-27

**Authors:** Francesca Vallese, Cristina Catoni, Domenico Cieri, Lucia Barazzuol, Omar Ramirez, Valentina Calore, Massimo Bonora, Flavia Giamogante, Paolo Pinton, Marisa Brini, Tito Calì

**Affiliations:** 1 Department of Biomedical Sciences, University of Padova, Padova, Italy; 2 Department of Biology, University of Padova, Padova, Italy; 3 Department of Neurobiology, Interdisciplinary Center for Neurosciences, Heidelberg University, Heidelberg, Germany; 4 Department of Morphology, Surgery and Experimental Medicine, Section of General Pathology, University of Ferrara, Ferrara, Italy; 5 Laboratory for Technologies of Advanced Therapies (LTTA), University of Ferrara, Ferrara, Italy; 6 Padova Neuroscience Center (PNC), University of Padova, Padova, Italy

**Keywords:** Imaging, Microscopy, Molecular engineering, Membrane biophysics, Cell signalling

## Abstract

Membrane contact sites between virtually any known organelle have been documented and, in the last decades, their study received momentum due to their importance for fundamental activities of the cell and for the subtle comprehension of many human diseases. The lack of tools to finely image inter-organelle proximity hindered our understanding on how these subcellular communication hubs mediate and regulate cell homeostasis. We develop an improved and expanded palette of split-GFP-based contact site sensors (SPLICS) for the detection of single and multiple organelle contact sites within a scalable distance range. We demonstrate their flexibility under physiological conditions and in living organisms.

## Abstract (web summary)

The authors have previously reported split-GFP-based contact site sensors (SPLICS) to document endoplasmic reticulum/mitochondria contact sites. Here they extend this work and develop a range of improved SPLICS sensors to detect single and multiple organelle contact sites at different distances.

## Introduction

Despite the fact that the general functions of organelles have been studied for decades, the concept of organelle contact sites has emerged in recent years. The molecular mechanisms by which defined domains of organelle membranes closely apposed and tethered without undergoing fusion events have been the object of intense investigation since it has been shown that the occurrence of numerous essential physiological processes is coordinated at the level of organelles contact sites^1^. Various protein complexes work in concert to perform specialized functions at organelle proximity such as binding, sensing, and transferring molecules^2^. Historically, the existence of sub compartmentalized endoplasmic reticulum (ER) membranes co-sedimented with the mitochondria^3^ was proven during cell-fractionation experiments, which are still considered the standard method when the presence/levels of specific players at the ER–mitochondria interface have to be investigated^4^. Other approaches, either based on electron microscopy (EM)^5–11^, on the co-localization of ER and mitochondrial markers^12–15^, on the use of pairs of primary antibodies against proteins on opposing membranes^16^, or on Förster resonance energy transfer (FRET)^17^ have been reported, but none of them has been applied to quantify the overall extent or geometry of the interface under physiologic conditions and in vivo. Novel tools to measure inter-organelle proximity have been recently developed^18–24^, nevertheless a reporter to easily image inter-organelle proximity over a range of distances in living cells still represents a major challenge. We have recently designed modular, split-green fluorescent protein (GFP) based contact site sensors (SPLICS) documenting the existence of at least two types of ER/mitochondria contact sites in human cells^19^. Furthermore, our SPLICS sensors enabled us to visualize contact sites in vivo in *D. rerio* Rohon Beard (RB) sensory neurons.

Here we describe a palette of expanded and improved single vector-based SPLICS sensors specifically designed to i) ensure equimolar production of the organelle-targeted GFP fragments, ii) expand the range of detection towards novel and unexplored short- and long-range contacts between disease-related organelles, and iii) simultaneously detect and quantify multiple heterotypic interactions in different spectral flavors both in vitro and in vivo.

## Results

### Design of four all-in-one SPLICS_S/L_-P2A reporters of organelle proximity

**Fig. 1:**
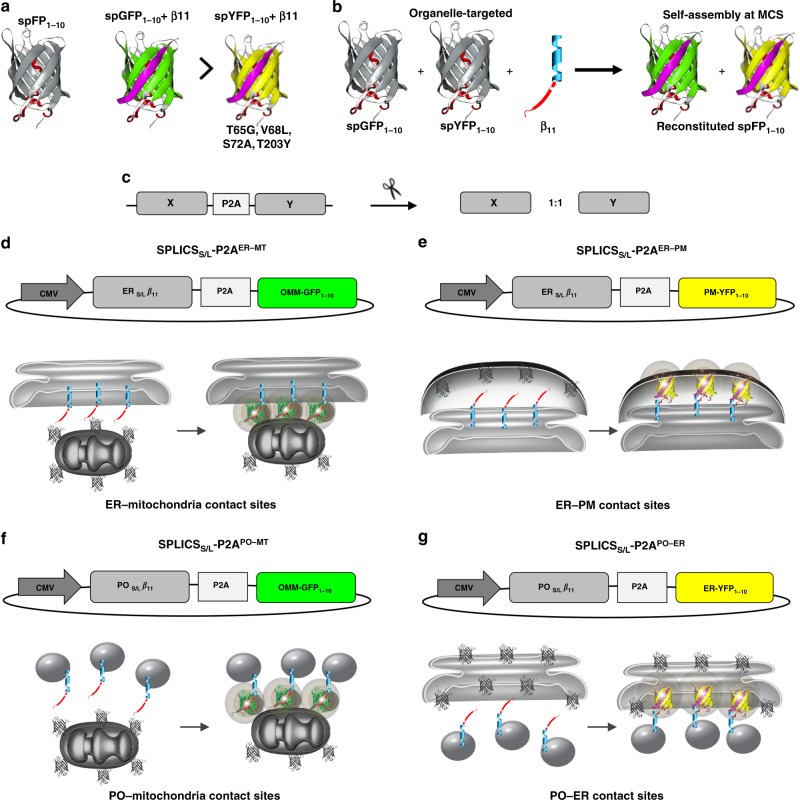
Design of the SPLICS_S/L_-P2A probes. **a** Cartoon representation of GFP/YFP SPLICS_S/L_-P2A probes. The cartoon also indicates the occurrence of reconstitution of the two spectral variants through association with the invariant strand β_11_. **b** Schematic representation of the targeted GFP/YFP SPLICS_S/L_-P2A chimeras. GFP/YFP protein was split in two non-fluorescent portions, G(Y)FP_1–10_ and β11 fragment that are targeted to outer face of organelles of interest. The complementation of targeted split-GFP at membrane contact sites was also shown in the cartoon. **c** Creation of a single vector for the equimolar expression of the targeted fragments through the insertion of the P2A peptide. **d**–**g** Schematic representations of the SPLICS_S/L_-P2A vectors. The β11_S/L_ coding sequence is cloned upstream the viral P2A peptide sequence, while the second cassette is occupied by the G(Y)FP_1–10_. **d** SPLICS_S/L_-P2A^ER–MT^ expression plasmids (ER_S/L_-β11 and OMM-GFP_1–10_). **e** SPLICS_S/L_-P2A^ER–PM^ expression plasmids (ER_S/L_-β11 and PM-YFP_1–10_). **f** SPLICS_S/L_-P2A^PO–MT^ expression plasmids (PO_S/L_-β11 and OMM-GFP_1–10_). **g** SPLICS_S/L_-P2A^PO–ER^ expression plasmids (PO_S/L_-β11 and ER-YFP_1–10_).

To build up scalable reporters for organelle contact sites we have first exploited the plasticity of the split-GFP system^25^. By taking advantage of the unique presence of the four color shifting point mutations in the GFP_1–10_ moiety, we converted the GFP_1–10_ in its yellow shifted variant yellow fluorescent protein (YFP)_1–10_ thus allowing the self-reconstitution of the two different spectral variants of the GFP_1–10_ through association with the same invariant β_11_ strand (Fig. 1a). The two non-fluorescent spectral GFP_1–10_ and YFP_1–10_ variants can be targeted, either alone or simultaneously, to the outer face of the organelles of interest along with the invariant and scalable β_11_ strand (Fig. 1b) to get either a GFP or a YFP signal upon reconstitution at the chosen organelle interface. The short- and long-range sensors that we have previously developed to detect interactions occurring over a range of ≈8–10 nm (SPLICS_S_) and ≈40–50 nm (SPLICS_L_), respectively^19^, were here improved by creating a bicistronic vector expressing equimolar amounts of the organelle-targeted GFP fragments through the insertion of a P2A peptide sequence^26^ (SPLICS_S/L_-P2A) (Fig. 1c). With these tools, by adding the opportune targeting sequence to the GFP_1–10_ or the YFP_1–10_ and the β_11_ strand moieties, we have generated a unique palette of SPLICS_S/L_ sensors for the detection of the majority of membrane contact sites, i.e., those between the ER and the mitochondria (Fig. 1d, SPLICS_S/L_-P2A^ER–MT^), the ER and the plasma membrane (PM) (Fig. 1e, SPLICS_S/L_-P2A^ER–PM^), peroxisomes (PO) and the mitochondria (Fig. 1f, SPLICS_S/L_-P2A^PO–MT^) and PO and the ER (Fig. 1g, SPLICS_S/L_-P2A^PO–ER^).

### Functional characterization of four all-in-one SPLICS_S/L_-P2A reporters

**Fig. 2:**
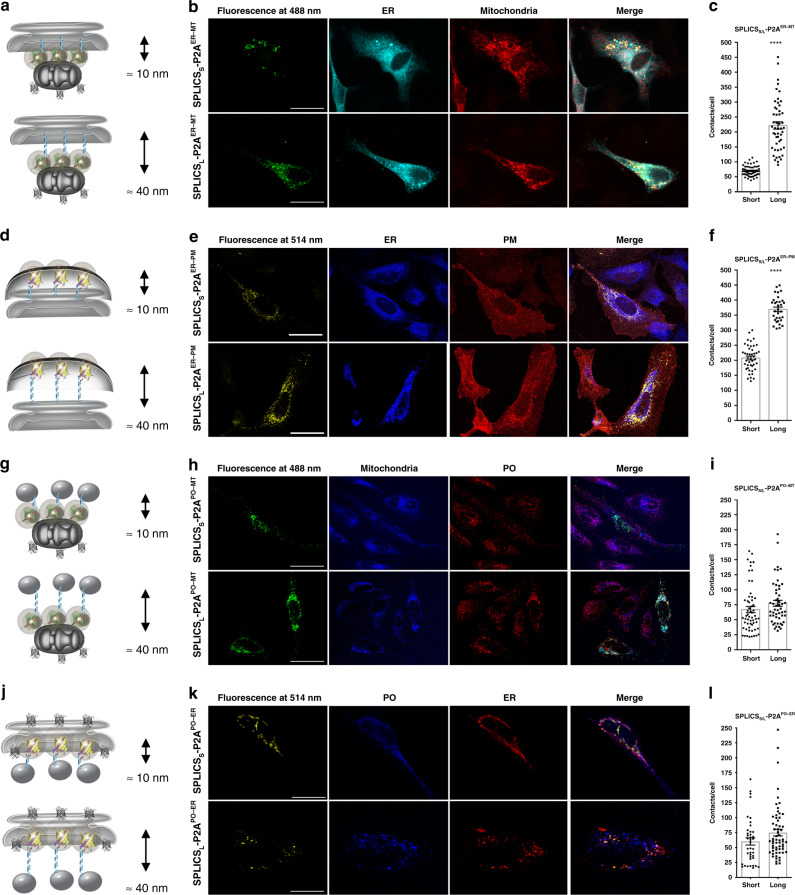
Functional characterization of the SPLICS_S/L_-P2A probes. **a**, **d**, **g**, **j** Cartoon showing the short- and long-range contact sites measured by the indicated reporters. **b**, **e**, **h**, **k** Representative images of HeLa cells expressing the indicated SPLICS_S/L_-P2A probes to identify the co-localization of GFP (488 nm) or YFP (514 nm) fluorescent dots with targeted organelles. Different markers are used to label organelles: red fluorescent protein (mCherry) targeted to PM, anti-KDEL antibody (ER), mtHSP60 (mitochondria) and anti-PMP70 antibody (PO). Confocal images were acquired at 405, 488, 514, and 588 nm excitation wavelength. Scale bar 25 µm. **c**, **f**, **i**, **l** Quantification of the indicated SPLICS_S/L_-P2A (short and long) contacts in HeLa cells. The SPLICS dots were quantified from the 3D rendering of a complete Z-stack (see Supplementary Fig. 2), mean ± SEM. The data were obtained from three independent transfections. (****P ≤ 0.0001 unpaired two-tailed *t*-test). Source data are provided as a source data file.

As mentioned, the reporters described above must be able to detect short- and long-range contact sites between the ER–mitochondria (Fig. 2a), the ER–PM (Fig. 2d), the PO–mitochondria (Fig. 2g), and the PO–ER (Fig. 2j) contact sites. To test their behavior, these single vector-based SPLICS sensors were expressed in HeLa cells giving rise to a typical dotty pattern that co-localized with the endogenous markers for the organelles of interest (Fig. 2b, e, h, k). Interestingly, quantification of the 3D rendered signal derived from integral Z-stack analysis (Fig. 2 and Supplementary Fig. 1) revealed statistically significant differences between short- and long-range contact sites at the ER–mitochondria (Fig. 2c) and the ER–PM interface (Fig. 2f), but not between the short and the long-range PO–mitochondria (Fig. 2i) and the PO–ER (Fig. 2l) interfaces (values Mean ± SEM: (c) SPLICS_S_-P2A^ER–MT^ 70.05 ± 2.28, *n* = 55 cells; SPLICS_L_-P2A^ER–MT^ 212.8 ± 10.78, *n* = 48 cells; (f) SPLICS_S_-P2A^ER–PM^ 208 ± 6, *n* = 44 cells; SPLICS_L_-P2A^ER–PM^ 369 ± 7, *n* = 32 cells; (i) SPLICS_S_-P2A^PO–MT^ 66.88 ± 5.14, *n* = 58 cells; SPLICS_L_-P2A^PO–MT^ 77.35 ± 5.04, *n* = 51 cells (l) SPLICS_S_-P2A^PO–ER^ 60.11 ± 6.01, *n* = 40 cells; SPLICS_L_-P2A^PO–ER^ 75 ± 5.67, *n* = 61 cells. Although these results suggest that while in the case of the ER interaction with the mitochondria or PM, contact sites occurring at different distances might well underpin different functions, as already suggested^19,27^, in the case of PO interaction with the ER or the mitochondria membranes no statistically significant heterogeneity is present, despite a trend towards an increase in the long-range interactions was observed. To better explore this aspect, two different PO membrane targeting sequences were tested for the generation of the SPLICS_S/L_-P2A^PO–MT^ probe: the fusion with the C-terminal transmembrane domain of the human ACBD5 protein^28^ and the addition of the PO membrane targeting sequence from the PEX3 protein^29^. Both the constructs were validated and found to be able to detect PO–MT contact sites (Supplementary Fig. 2a, b, c). Interestingly, a statistically significant difference between the short- and the long-range PO–MT contacts is detected by the PEX3 targeted constructs. Considering that the targeting of Pex3 could change during different phases of organelle biogenesis/maturation^30,31^, for further experiments we have decided to use the reporter containing the C-terminal domain of the human ACBD5 protein as targeting sequence and further check for possible heterogeneity in PO–MT and PO–ER contact sites with a reporter able to detect both of them simultaneously in the same cell (see below).

**Fig. 3:**
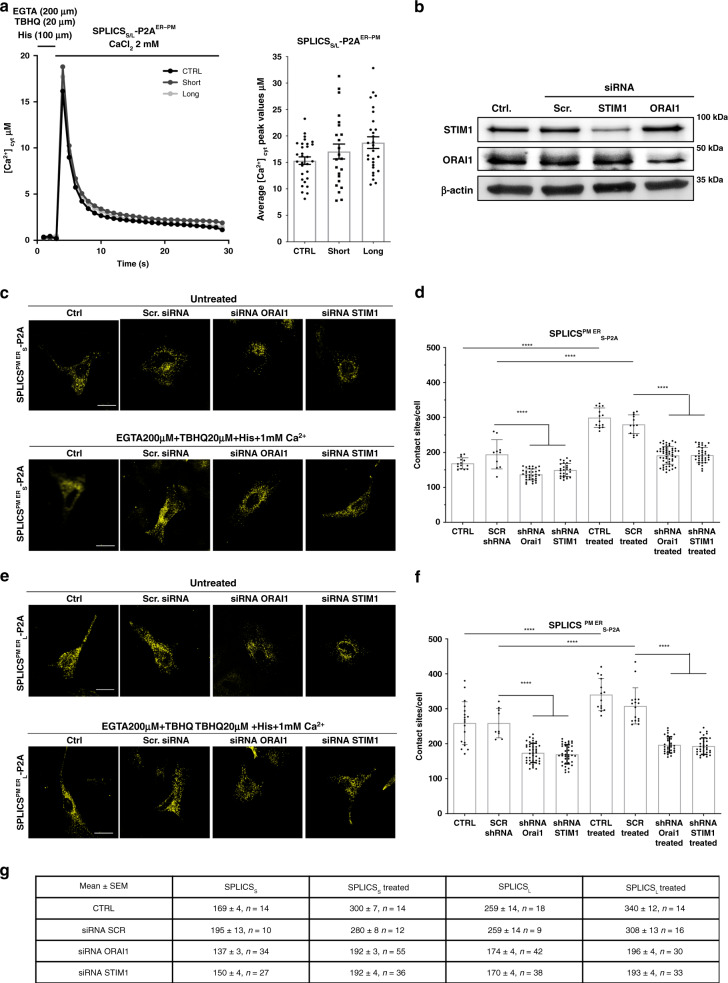
Deep proof-of-concept characterization of the ER–PM reporter. **a** Cytosolic Ca^2+^ transients (left) and quantification (right) upon induction of the ER Ca^2+^ depletion in cells overexpressing SPLICS_S_-P2A^ER–PM^ and SPLICS_L_-P2A^ER–PM^ probes. To induce the ER Ca^2+^ depletion cells were treated with 20 µM 2,5-tertbutylhydroquinone (THBQ), 100 μM histamine, and 200 μM EGTA. Where indicated 2 mM CaCl_2_ was then applied to induce Ca^2+^ influx. The traces are the media of at least 15 independent measurements obtained from two independent transfections, mean ± SEM. **b** Expression levels of STIM1 and ORAI1 proteins in mock (Ctrl), siRNA scramble or siRNA STIM1, or SiRNA ORAI1 treated cells were analyzed by Western blotting with an anti-STIM1 or anti-ORAI1 antibodies. Equal amount of total loaded lysate was verified by incubation with anti-β actin antibody. Effect of STIM1/ORAI1 silencing and the ER Ca^2+^ depletion on short **c** and long **e** range contacts between the ER and PM. Representative microscope single plane images of HeLa cells expressing the SPLICS_S/L_-P2A^ER–PM^ untreated (top) or treated for 5 min (bottom) with 200 μM EGTA, 20 µM THBQ, 100 μM histamine, and then for additional 5 min with 1 mM CaCl_2_ supplemented to KRB. Scale bar 25 µm. Quantification of SPLICS_S_-P2A^ER–PM^ **d** and SPLICS_L_-P2A^ER–PM^ **f** contacts by 3D rendering of complete Z-stacks, mean ± SEM. The data were obtained from three independent transfections (****p ≤ 0.0001 unpaired two-tailed *t-*test). **g** Mean ± SEM values of the number of PM–ER contacts in control conditions, upon SOCE activation and upon siRNA scramble or siRNA STIM1 or SiRNA ORAI1 incubation. Source data are provided as a source data file.

As a proof of concept experiment, the flexibility of the described sensors in terms of ability to detect subtle changes in the number of contact sites has been further tested in one of the most established and extremely dynamic pathway known so far, the STIM1–ORAI1 microdomain occurring at the ER–PM interface. A sagittal projection over a complete Z-stack confirmed that the SPLICS_S_-P2A^ER–PM^ fluorescence signals indeed co-localize in the vicinity of the cellular boundaries (Supplementary Fig. 3). We then verified whether expression of the SPLICS_S/L_-P2A^ER–PM^ reporter could affect cytosolic Ca^2+^ transients induced by Ca^2+^ entry from the extracellular ambient evoked upon intracellular stores depletion. To this aim, aequorin-based measurements of cytosolic Ca^2+^ transients were performed to evaluate whether unwanted non-physiological contact sites between the ER and PM eventually could be induced by the expression of the SPLICS_S/L_-P2A^ER-PM^ reporter and thus increase Ca^2+^ entry. Figure 3a, b shows that no major differences were observed between control cells (exclusively expressing cytosolic aequorin) and the cells expressing also the reporters (values Mean ± SEM: [Ca^2+^]_cyt_ μM 15.34 ± 0.69 *n* = 30 for control cells; 17.06 ± 1.41 *n* = 23 for SPLICS_S_ overexpressing cells; 18.73 ± 1.09 *n* = 28 for SPLICS_L_ overexpressing cells). Once verified that no major impact on the endogenous Ca^2+^ fluxes between the ER and PM is induced by the reporter, downregulation of STIM1 or ORAI1 was performed by shRNA in order to test the flexibility of the sensor to detect changes in the ER–PM interface. HeLa cells were transfected with the SPLICS_S/L_-P2A^ER–PM^ reporter and with the shRNAs for the downregulation of the STIM1 or ORAI1 proteins, the main components of the Store Operated Calcium Entry pathway (SOCE). Western blotting with an anti-STIM1 or anti-ORAI1 antibodies in mock (Ctrl), siRNA scramble or siRNA STIM1 or SiRNA ORAI1 treated cells verified that the expression of the STIM1 and ORAI1 proteins was indeed reduced (Fig. 3b). The detection of the ER–PM contact sites was then performed and analyzed either under basal condition or after stores Ca^2+^ depletion by using both the SPLICS_S_-P2A^ER–PM^ (Fig. 3c) and the SPLICS_L_-P2A^ER–PM^ (Fig. 3e) reporters in order to measure short- and long- range contact sites.

Interestingly, the number of short- (Fig. 3d) and long-range (Fig. 3f) ER–PM contact sites increased upon SOCE activation and decreased upon ORAI1 or STIM1 downregulation as clearly shown from the quantification analysis (see Fig. 3g and Supplementary Fig. 4a, b for the 3D rendered Z-stacks), suggesting that our reporters are well able to detect STIM1–ORAI1 dependent ER–PM contact sites taking place in the range between 10 nm and 50 nm^32^. Moreover, the consistent number of contact sites detected under basal conditions (i.e., control untreated) indicates that our probes are able to reveal the presence of ER–PM interaction occurring both at long and short distance independently from SOCE activation.

### Generation of dual reporters

**Fig. 4:**
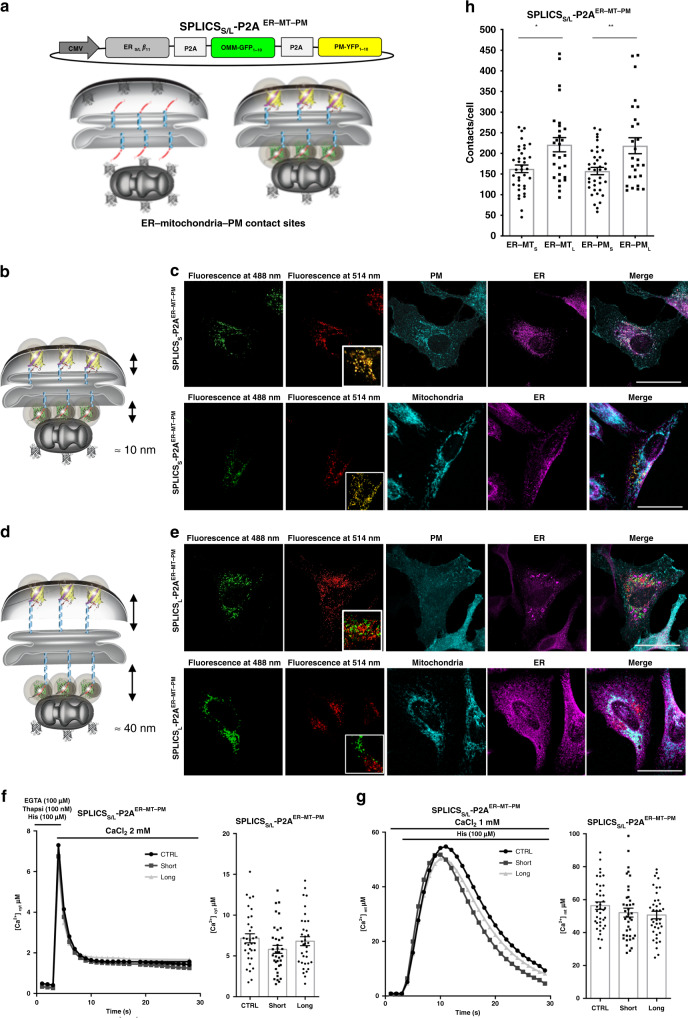
Functional characterization of SPLICS_S/L_ sensors to detect the ER–mitochondria and the ER–PM interfaces simultaneously. **a** Schematic representation of the constructs SPLICS_S/L_ P2A^ER–MT–PM^ able to detect short (8–10 nm) and long (40–50 nm) range contact sites between the ER–mitochondria and the ER–PM simultaneously in different spectral variants. The cartoon shows the approach used to design the SPLICS reporters and indicates that the complementation of either short- and long-range ER-β_11 fragments_ with OMM-GFP_1–10_ and PM-YFP_1–10_ will reveal a florescence signal at the level of the contact sites. **b**, **d** Cartoon showing the short- and long-range contact sites measured by the indicated reporters. Confocal images of HeLa cells transfected with SPLICS_S_ P2A^ER–MT–PM^ **c** and SPLICS_L_ P2A^ER-MT-PM^ **e** showing the appearance of fluorescent “dots” upon excitation at 488 and 514 nm wavelengths confirming the complementation with GFP_1–10_ (in green) and YFP_1–10_ (in pseudocolour, red) at the ER–MT and the ER–PM contact sites, respectively. The co-localization of the SPLICS_S/L_ P2A^ER–MT–PM^ reporters with PM-cherry (in cyan) as plasma membrane marker and with an endogenous marker of ER (KDEL, in magenta) (upper panels) and with mitochondria (TOM20, in cyan)/ER (KDEL, in magenta)/(lower panels) is shown. Scale bar 25 µm. Cytosolic **f** and mitochondrial **g** Ca^2+^ transients in cells overexpressing SPLICS_S_-P2A^ER–MT–PM^ and SPLICS_L_-P2A^ER–MT–PM^ probes, mean ± SEM. To induce ER Ca^2+^ depletion cells were incubated for 5 min in KRB supplemented with 100 nM thapsigargin, 100 μM histamine, 100 μM EGTA and then exposed, where indicated, to KRB containing 2 mM CaCl_2_. The traces are obtained from at least three independent transfections, values. **h** Quantification of the ER–mitochondria and the ER–PM (short and long) contacts in HeLa cells. The SPLICS dots were quantified from the 3D rendering of a complete Z-stack, mean ± SEM. The data were obtained from three independent transfections. (*P = 0.013, **P = 0.0052 one-way ANOVA). YFP emitted fluorescence is shown as pseudocolour in red to better appreciate in yellow the co-localization with the green signal of GFP. Source data are provided as a source data file.

**Fig. 5:**
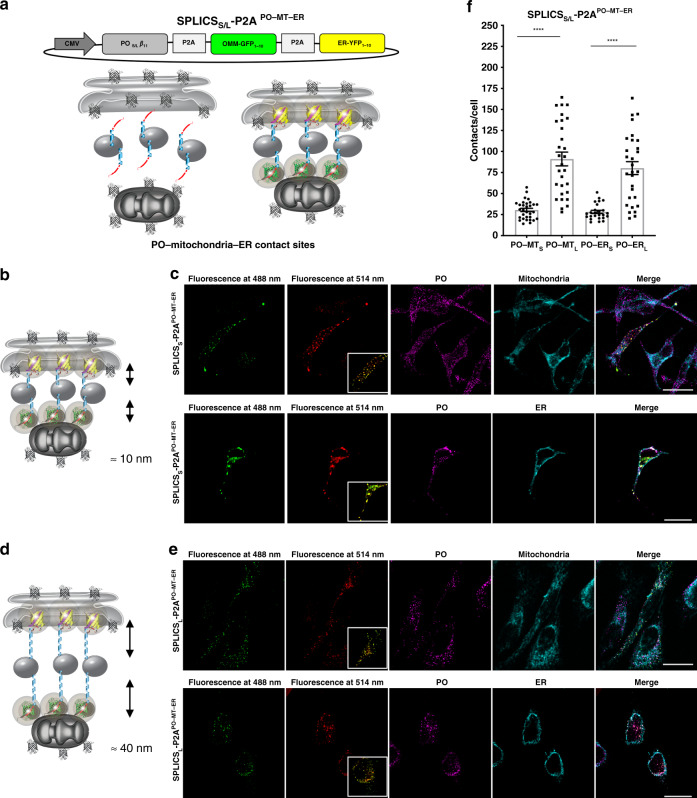
Functional characterization of SPLICS_S/L_ sensors to detect the PO–mitochondria and the PO–ER interfaces simultaneously. **a** Schematic representation of the constructs SPLICS_S/L_ P2A^PO–MT–ER^ able to detect short (8–10 nm) and long (40–50 nm) range contact sites between the ER–mitochondria and the ER–PM simultaneously in different spectral variants. The cartoon shows the approach used to design the SPLICS reporters and indicates that the complementation of either short- and long-range PO-β_11 fragments_ with OMM-GFP_1–10_ and ER-YFP_1–10_ will reveal a florescence signal at the level of the contact sites. **b**, **d** Cartoon showing the short- and long-range contact sites measured by the indicated reporters. Confocal single plane images of HeLa cells transfected with SPLICS_S_ P2A^PO–MT–ER^ **c** and SPLICS_L_ P2A^PO–MT–ER^ **e** showing the appearance of fluorescent “dots” upon excitation at 488 and 514 nm wavelengths confirming the complementation with GFP_1–10_ (in green) and YFP_1–10_ (in pseudocolour, red) at the PO–MT and the PO–ER contact sites, respectively. The co-localization of the SPLICS_S/L_ P2A^PO–MT–ER^ reporters with endogenous markers of PO (PMP70)/mitochondria (TOM20) (upper panels) and PO (PMP70)/ER (KDEL) (lower panels) is shown. Scale bar 25 µm. **f** Quantification of the PO–mitochondria and the PO–ER (short and long) contacts in HeLa cells. The SPLICS dots were quantified from the 3D rendering of a complete Z-stack, mean ± SEM. The data were obtained from three independent transfections. (****P ≤ 0.0001 unpaired two-tailed *t*-test). Source data are provided as a source data file.

Once verified that the same short and long strand β_11_ was indeed able to reconstitute the fluorescence of either the GFP_1–10_ or the YFP_1–10_ moiety, we proceeded with generation of a SPLICS sensor able to detect short- and long-range membrane contact sites occurring between different organelle interfaces simultaneously within the same cell. To this aim we have created an all-in-one vector for the simultaneous detection of the short- or long-range ER–MT and ER–PM interactions (SPLICS_S/L_-P2A^ER–MT–PM^) (Fig. 4) as well as of the PO–MT and PO–ER interactions (SPLICS_S/L_-P2A^PO–MT–ER^) (Fig. 5). These tools have been generated by fusing in tandem the three GFP portions intercalated by two P2A sequences (i.e., the ER-targeted β_11_ strand, the OMM-GFP_1–10_ and the PM-YFP_1–10_ for the SPLICS_S/L_-P2A^ER–MT–PM^ and the PO-targeted β_11_ strand, the OMM-GFP_1–10_ and the ER-YFP_1–10_ for the SPLICS_S/L_-P2A^PO–MT–ER^) to ensure equimolar expression of each organelle-targeted fragment (Figs. 4a and 5a). The abovementioned constructs, designed to detect short ER–mitochondria and ER–PM or long ER–mitochondria and ER–PM contact sites simultaneously (Fig. 4b, d), were expressed in HeLa cells and co-localization with endogenous organelle markers was performed to establish whether a specific signal could be detected at the specific membrane interface. Specifically, markers of ER (anti-KDEL antibody) with either PM (PM-targeted mCherry) (Fig. 4c, e upper panels) or mitochondria (anti-TOM20 antibody) (Fig. 4c, e lower panels) for the SPLICS_S_-P2A^ER–MT–PM^ and SPLICS_L_-P2A^ER–MT–PM^ were utilized, respectively. All co-localizations were performed by double immunofluorescence and imaged by confocal microscopy (see also Supplementary Fig. 5 for a sagittal section). Western Blot analysis with an anti-GFP/YFP antibody was also performed on HeLa cells expressing the double reporters involving the ER, mitochondria and PM (SPLICS_S/L_-P2A^ER–MT–PM^) and revealed no differences in the amount and in the correct and efficient cleavage of the protein chains inside the cells in comparison with the single reporters (SPLICS_S/L_-P2A^ER–MT^, SPLICS_S/L_-P2A^ER–PM^) (see Supplementary Figs. 6 and 7 for the expected molecular weights of the GFP sensors). Intriguingly, as shown in Fig. 4c the GFP_1–10_ and the YFP_1–10_ signals overlapped almost completely when the short-range construct SPLICS_S_-P2A^ER–MT–PM^ was transfected. However, co-localization of the two signals was almost absent when the long-range contacts were imaged with the SPLICS_L_-P2A^ER–MT–PM^ probe (Fig. 4e), suggesting the possibility that membrane interactions that occur in the short range of distance could involve more than two organelles, i.e., the formation of triple junctions, while those occurring over longer distances do not. The measurements of cytosolic Ca^2+^ transients induced by Ca^2+^ influx after intracellular stores depletion (Fig. 4f) and of mitochondrial (Fig. 4g) Ca^2+^ transients induced by cell stimulation with an inositol 1,4,5 trisphosphate (Insp3) linked agonist showed that no significant changes were detected in Hela cells expressing the SPLICS_S/L_^ER–MT–PM^ sensors in respect with mock cells (values Mean ± SEM: [Ca^2+^]_cyt_ μM 7.16 ± 3.2 for control cells, *n* = 33; 5.8 ± 2.9 for SPLICS_S_ overexpressing cells, *n* = 36; 6.8 ± 3.2 for SPLICS_L_ overexpressing cells, *n* = 35; [Ca^2+^]_mt_ μM 56.40 ± 14.23 for control cells, *n* = 39; 52.13 ± 17.0 for SPLICS_S_ overexpressing cells, *n* = 39; 50.67 ± 13.9 for SPLICS_L_ overexpressing cells, *n* = 38), thus indicating that the Ca^2+^ fluxes regulated by the ER–PM and the ER–mitochondria interface were not modified by the presence of the reporter and suggesting that no artificial contacts were indeed induced by the expression of these sensors. Quantification of the extent of short and long ER–mitochondria/ER–PM contact sites (Fig. 4h) from the 3D rendered signal of integral Z-stacks (Supplementary Fig. 8) revealed that the number of ER–MT and ER–PM contact sites is unexpectedly similar and that the contacts at the long distance are more frequent than those at the short one, as already documented by the single reporters in Fig. 2 (values Mean ± SEM: ER–MT_S_ 162.7 ± 9.16, *n* = 36 cells; ER–MT_L_ 221.1 ± 17.19, *n* = 28 cells; ER–PM_S_ 157.4 ± 8.93, *n* = 37 cells; ER–PM_L_ 218.7 ± 19.39, *n* = 27 cells).

As anticipated above, sensors for the detection of short PO–Mitochondria/short PO–ER or long PO–Mitochondria/long PO–ER contact sites simultaneously have been also designed and tested (Fig. 5b, d). Double immunofluorescence analysis with markers of PO (anti-PMP70 antibody) with either mitochondria (anti-mtHSP60 antibody) or ER (anti-KDEL antibody) were also assessed for the SPLICS_S_-P2A^PO–MT–ER^ (Fig. 5c upper panels) and the SPLICS_L_-P2A^PO–MT–ER^ (Fig. 5e lower panels) sensors, respectively. Interestingly a good but not complete degree of co-localization between PO–MT and PO–ER contacts (see the insets in Fig. 5c, e and Supplementary Fig. 9) was observed, suggesting that the short- and long-range contacts formed by PO and the mitochondria and PO and the ER are differently regulated and might be engaged in different cellular functions. Quantification of the extent of short and long PO–mitochondria/PO–ER contact sites (Fig. 5f) from the 3D rendered signal of integral Z-stacks (Supplementary Fig. 10) revealed that PO associations with the mitochondria are quantitatively similar to those with the ER (values Mean ± SEM: PO–MT_S_ 30.52 ± 1.98, *n* = 31 cells; PO–MT_L_ 91.12 ± 8.13, *n* = 29 cells; PO–ER_S_ 28.16 ± 1.89, *n* = 25 cells; PO–ER_L_ 80.28 ± 7.62, *n* = 29 cells. ****P ≤ 0.0001). This feature, however, could be dependent on the cell type used since other studies have reported a much higher frequency of PO–ER contacts in COS7 cells^33^. However, a statistically significant difference can be clearly detected between short- and long-range PO–MT and PO–ER contacts, in line with the trend towards an increase, but not statistically significant, reported in Fig. 2 with the single reporters. This aspect deserves further investigation but the possibility to investigate, simultaneously, in the same cells PO–MT and PO–ER contacts is intriguing and can reveal high complexity in the PO relationship with different organelles.

### In vitro and in vivo detection of dynamic contact sites

**Fig. 6:**
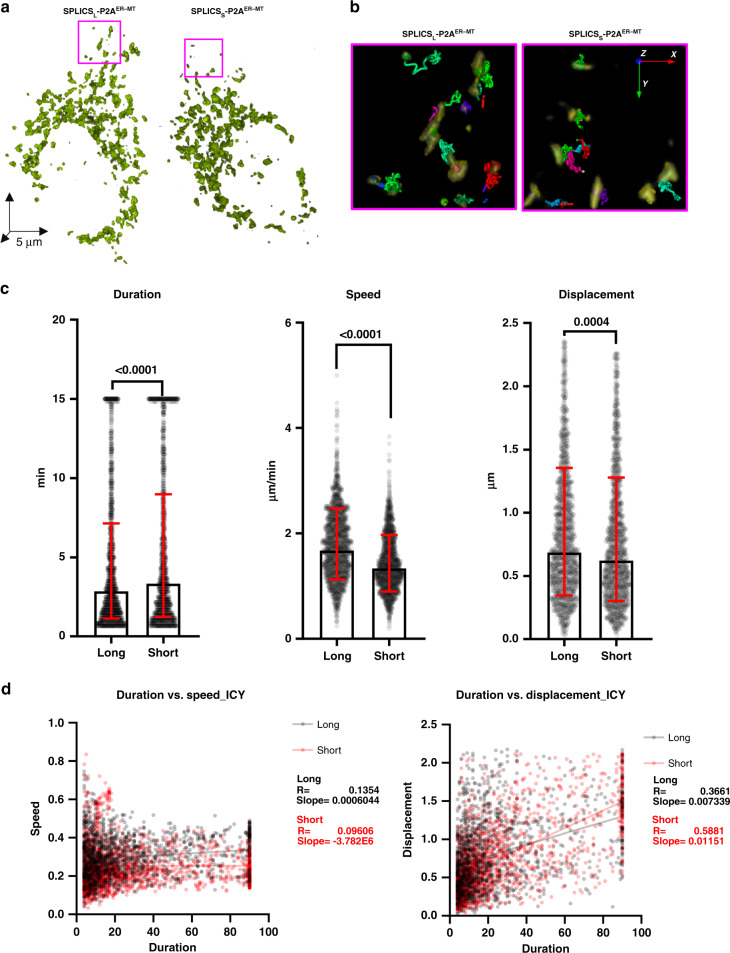
ER–mitochondria contact sites are dynamic structures in cultured cells. **a** Representative rendering of HeLa cells expressing SPLICS_S_ P2A^ER–MT^ (left panel) or SPLICS_L_ P2A^ER–MT^ (right panel). **b** Representative isosurface rendering of tracks obtained by the Icy method of cropped areas from **a**. Tracks are color coded to represent track ID. **c** Tracks quantitative analysis obtained by the Icy method of track duration (*n* = 1645 long and 1504 short tracks from six cells), speed (*n* = 1671 long and 1502 short tracks from six cells) and displacement (*n* = 1461 long and 1395 short tracks from six cells), geometric mean ± geometric SD. **d** Duration/speed and Duration/displacement correlation obtained by the Icy method. The Parson coefficient and the slope are indicated for the long- and short-range ER–mitochondria contact sites, respectively. Data shown are the result of three independent transfections. (Mann–Whitney or Kolmgorov–Smirnov tests). Source data are provided as a source data file.

**Fig. 7:**
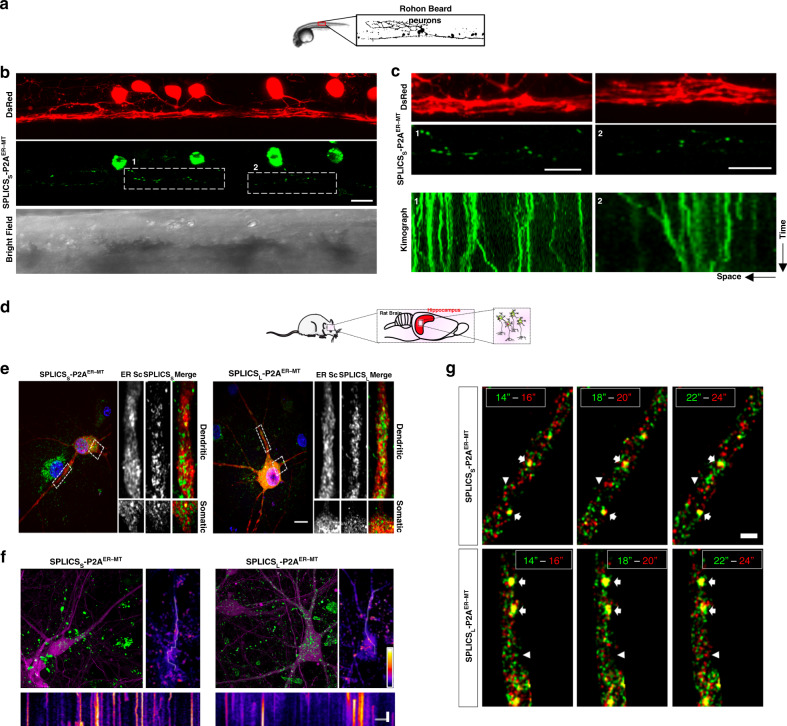
ER–mitochondria contact sites are dynamic structures in vivo. **a** Live imaging of ER–mitochondria contact sites in zebrafish RB neurons. **b** Representative confocal images of SPLICS_S_-P2A^ER–MT^ in RB neurons of 24 hpf s1102t:GAL4 living embryos injected with the pT2-DsRed-UAS-SPLICS_S_-P2A^ER–MT^ construct. Rostral is on the right, dorsal on the top. BF Bright Field. **c** Magnification of the areas shown in **b** and kymographs obtained by time-lapse recordings of the indicated region of the cell. The width of the kymograph represents the length of the axon recorded during the time-lapse, whereas the height reflects the elapsed time. Scale bar: 15 μm. **d** Monitoring of ER–mitochondria contacts in rat hippocampal neurons by SPLICS_S/L_-P2A^ER–MT^. **e** ER–mitochondria wide and narrow contact sites are distributed throughout the entire somatodendritic and axonal compartments in hippocampal neurons. Rat hippocampal neurons were co-transfected on DIV 11 with the endoplasmic reticulum marker ER-mScarlet (ER-Sc) together with either the short- (8–10 nm) or long-range (40–50 nm) sensors, SPLICSs-P2A^ER–MT^ (left) or SPLICS_L_- P2A^ER–MT^ (right), respectively. On DIV 12, neurons were incubated with Hoechst 33258 and fixed before confocal imaging. High-resolution Z-stacks were acquired in order to visualize nuclei (blue), ER (red), and ER–mitochondria contact sites (green). Selected dendritic (up) and somatic (bottom) regions are zoomed and shown on the right for a detailed view. Scale bar represents 10 µm. **f** Short- or long-range SPLICS_S/L_-P2A^ER–MT^ (green) were transduced on DIV 7 with rAAVs to convey sparse expression of RFP (magenta)^34^ and on DIV 11 neurons were transferred to an imaging chamber containing CO_2_-independent CICM medium and a Z-stack was acquired in a spinning disk microscope and shown as Z-stack maximum projection for a general overview (upper-left panels). By using a single plane, a time series of the SPLICS_L/S_ structures was acquired during 4 min at 0.5 Hz. Standard deviation analysis was applied to the image (upper-right panels, scale bars represent 10 μm and the color table), which was used to identify mobile structures and to choose the path for its visualization in a kymograph (lower panels). The scale bars represent 60 s (vertical) and 10 μm (horizontal). **g** ER–mitochondria contact sites dynamics suggest high turnover rates in hippocampal neurons under basal unstimulated conditions. Rat hippocampal neurons were infected on DIV 7 with rAAVs to convey expression of the long- or short-range SPLICSL/S (green and red). On DIV 11, neurons were transferred to an imaging chamber containing CO_2_-independent medium and a time series was acquired in a spinning disk microscope at 0.5 Hz (same neurons shown in Fig. 7f). Three examples of two consecutive time points spanning 2 s in between are shown in the combined images, where the green and red colors indicate the initial and final time points, respectively. Arrows indicate hot-spots of long-lived SPLICS^ER–MT^ signals and arrowheads indicate short-lived SPLICS^ER–MT^. The scale bar represents 2 μm. (The Image in panel d is a previously created element from Servier Medical Art https://smart.servier.com under the Creative Commons Attribution 3.0 Unported License).

Organelles are dynamic structures able to adapt to rapid changes in shape and function to cope with multiple cellular needs. As a consequence, contact sites should also possess a dynamic behavior in time and space to properly sustain defined functions at the specific interfaces. We have thus decided to test our sensors and their dynamic nature in vitro and in vivo by performing time-resolved experiments. Figures 6 and 7 show this kind of experiments with SPLICS_S/L_-P2A^ER–MT^ in three different models. To this aim SPLICS_S/L_-P2A^ER–MT^ were transfected in cultured HeLa cells (Fig. 6) or co-transfected along with a cytosolic mCherry in Zebrafish RB sensory neurons (Fig. 7a–c) or in mouse hippocampal neurons (Fig. 7d–f). In cultured HeLa cells, three different detection and tracking algorithms couples reported that the long-range ER–mitochondria interactions have lower persistence in time (lifetime, Fig. 6a–c and Supplementary Fig. 11a, b) but higher displacement (meant as the distance between starting and ending point of the track) as well as movement speed compared to the short range ER–mitochondria interactions (values left panel, geometric mean ± geometric SD: ER–MT_S_ 2.543 ± 2.275, *n* = 1269 tracks from six cells; ER–MT_L_ 2.207 ± 2.075, *n* = 1404 tracks from six cells; track speed, middle panel, geometric mean ± geometric SD: ER–MT_S_ 1.1.334 ± 1.478, *n* = 1269 tracks from six cells; ER–MT_L_ 1.672 ± 1.483, *n* = 1404 tracks from six cells; track displacement, right panel, geometric mean ± geometric SD: ER–MT_S_ 0.6068 ± 2.021, *n* = 1395 tracks from six cells; ER–MT_L_ 0.6699 ± 1.956, *n* = 1461 tracks from six cells). This indicates that long-range contacts remodel to a greater extent. The duration/speed and duration/displacement analysis performed with three different detection and tracking algorithms (Fig. 6d and Supplementary Fig. 11c, d) also showed a more pronounced behavior for the short-range contacts, indicative of a longer half-life, a slower speed, and a higher probability to move.

Along the same line, high-resolution images were acquired over time in a confocal microscope to visualize ER–mitochondria contact sites that appeared either in the soma or distributed in the axons of RB neurons marked in red (Fig. 7a, b). Kymographs from the time-lapse live-imaging movies were generated (Fig. 7c) and revealed a dynamic nature of the interface between these organelles in time and space (see also Supplementary Movies 1-6 for the short-range ER–PM contact sites in HeLa cells). SPLICS_S/L_-P2A^ER–MT^ expression in mouse hippocampal neurons further confirmed this behavior (Fig. 7d–f). Either short or long ER–mitochondria contact sites could be visualized throughout the entire somatodendritic and axonal compartments (Fig. 7e). To improve the efficiency of the SPLICS sensors expression, neuronal cultures were transduced with recombinant adeno-associated virus (rAVVs) carrying the sequences of both SPLICS_S/L_-P2A^ER–MT^ and the Supernova system^34^ to achieve sparse labeling of individual neurons, identified and chosen for visualization in a kymograph (Fig. 7f). Time lapse analysis performed under those conditions by acquiring three examples of two consecutive time points spanning 2 s in between also revealed that little and fainter structures are highly dynamic, whereas some of the larger spots tend to be very stable, but not static, (Fig. 7g, where the green and red colors indicate the initial and final time points, respectively, while hot-spots of long-lived SPLICS^ER–MT^ and short-lived SPLICS^ER–MT^ signals are indicated by arrows and arrowheads, respectively). Although to a lesser extent compared to the results in RB neurons, these results confirmed the dynamic nature of the ER–mitochondria contact sites and, importantly, demonstrated that contact sites can be dynamically monitored to understand whether and how they are modulated in time and space and to unveil the mechanism(s) by which they can travel within the cell under physiological and pathological conditions.

## Discussion

Here we report an all-in-one palette of SPLICS sensors to image either known or inter-organelle interactions occurring at different distances that could never be detected so far. Short- and long-range ER–mitochondria, ER–PM, PO–Mitochondria, and PO–ER contact sites can be detected in an easy, single-step and semi-automated manner. We also demonstrate that SPLICS sensors are suitable to monitor inter-organelle interactions in human cells, in primary hippocampal neurons and in vivo in zebrafish embryo RB neurons by simple fluorescence microscopy methods.

The SPLICS probes described here can reveal short- and long-range ER–mitochondria interactions without affecting endogenous functions of the organelles, e.g., the monitoring of Ca^2+^ fluxes is a proof of concept for this. Because this parameter is paramount in defining the function of organelle contact sites, the SPLICS reporters are amenable to simultaneously image and functionally address changes in several types of heterotypic membrane apposition in living cell cultures and in vivo. An advantage of SPLICS relies not only on its modularity, but also on the improved properties in terms of folding efficiency and brightness deriving from the superfolder variant of the GFP used, that confers to the fluorescent signal a high stability and a high threshold over background. Two versions of SPLICS, one engineered to detect the occurrence of narrow (≈8–10 nm), and a second to image wide (≈40–45 nm) organelle interactions^6,12^ were generated. By using these two tools, we were able to provide some insights into the biology of the organelle interface.

By exploiting the flexibility of the GFP protein we were also able to detect narrow and wide interactions occurring between different organelle couples within the same cell in different spectral flavors, i.e., simultaneously. This engineering work ended up with the generation of a class of sensors tested for their ability to detect simultaneous organelle interactions occurring between the ER–mitochondria and the ER–PM (namely the SPLICS_S/L_-P2A^ER–MT–PM^) and occurring between PO–Mitochondria and PO–ER (SPLICS_S/L_-P2A^PO–MT–ER^) in different spectral variants making them unique tools to explore the nature of these organelle interfaces under different physiological and pathological conditions in vitro and in vivo. Eventually this approach can be extended to explore additional tripartite contact sites^35,36^. One final point deserves mention: organelles are dynamic; thus, organelle contact sites must also be dynamic. Unfortunately, the monitoring of the dynamic nature of a “single” contact in time and space is not so obvious. We have demonstrated here that under selected conditions the contact sites between the ER and the mitochondria are dynamic structures. This is important since the contact sites dynamic might well be affected under different physiopathological conditions. It should be mentioned, however, that this dynamic nature is intended in terms of contact movement, since we are still unable at this stage to clearly define whether at a given organelle interface there is enough force to allow the formation/dissociation of a contact site. At the same time we also do not know whether the movement of the contact is due to the mitochondria movement sliding on the ER surface, the opposite or to the existence of a defined machinery evolved to coordinate the movement of the contact site within two opposing membranes, additional experiments should clarify these important points. Last, but not least, there are important technical advantages in the use of the SPLICS sensors which consist in the transfection of one single vector, in the improved folding efficiency, in the high signal-to-noise ratio and in the possibility to automate the quantification process that makes the SPLICS reporters ideal for high-throughput screening of compounds or intracellular modulators able to modulate a specific organelle’s interface.

In conclusion, in the present report we described the generation and the validation of an expanded and improved palette of reporters for the detection of single and multiple organelle contact sites that could not be otherwise detected. Furthermore, we also exploited their flexibility in vitro and in vivo. Defects in many proteins that have been described to participate to membrane contact sites regulation are linked to different diseases such as Alzheimer’s Disease, Parkinson’s Disease, hereditary spastic paraplegia, amyotrophic lateral sclerosis, Charcot–Marie–Tooth disease, and retinal dystrophy^37^. However, whether a defect in the membrane contact sites function is involved in the development of these diseases, directly or indirectly, remains unknown. In this scenario, the possibility to visualize and quantify contact sites in a simple one-step manner represents an opportunity for high-scale screening of drugs that modulate contact sites (eventually also for therapeutic purposes) or for following contact sites in disease models over time. Last but not least, the ability to perform multicolor imaging to monitor simultaneously different (narrow and wide or between two competing organelles) contact sites within the same cell both in vitro and in living organisms will be of pivotal importance to uncover the mechanisms of organelle contact sites formation/stabilization and their dynamic nature.

## Methods

### Hippocampal cultures

Hippocampal neurons from new-born Sprague-Dawley rats were obtained by dissociation at 37 °C for two 30-min periods in a medium containing 1.4 mM Hepes (pH 7.4), 73.6 mM Na_2_SO_4_, 27 mM K_2_SO_4_, 15.2 mM MgCI_2_, 0.23 mM CaCI_2_, 1 mM sodium kynurenate, 18 mM glucose, and 0.0011% phenol red; the glucose concentration in L-15 CO_2_ growth medium was 30 mM. The Ca^2+^ and Mg^2+^ concentrations in L-15 CO_2_ growth medium were 0.9 mM and 1.3 mM, respectively. Cells were plated on poly(D-lysine)-and laminin-coated 60-mm tissue culture dishes at a density of 0.05–0.06 hippocampi per square centimeter and were grown at 37 °C in a humidified atmosphere consisting of 5% CO_2_ plus 95% air. To inhibit proliferation of non-neuronal cells, 2.4 μM cytosine-p-D-arabinofuranoside was added to the cultures 1–2 days after plating^38,39^. Experiments were done after a culturing period of 7–12 DIV. DNA transfections were done on DIV 11 by using Lipofectamine 2000 (Invitrogen, San Diego, CA)^40^, and fixed for analysis on DIV 12. For the live cell imaging of the SPLICS sensors dynamics, neurons were transduced with rAAVs on DIV 7 and on DIV 11 transferred to an imaging chamber containing CO_2_-independent culture medium (CICM) consisting of 140 mm NaCl, 2.5 mM KCl, 1.0 mM MgCl_2_, 2.0 mM CaCl_2_, 10.0 mM Hepes, 1.0 mM glycine, 35.6 mM D-glucose, and 0.5 mM C_3_H_3_NaO3. Images were acquired in a Nikon Ti inverted microscope equipped with a Yokagawa CSU-X1, using a Plan Apo VC 100x NA 1.4 oil immersion objective. Recombinant adeno-associated viruses (rAAVs) viral particles driving the expression of Supernova and SPLICS_S/L_ were produced and purified^41^.

### Cloning and fusion plasmid construction

SPLICS_S/L_-P2A^ER–PM^, SPLICS_S/L_-P2A^ER–MT–PM^, SPLICS_S/L_-P2A^PO–MT^, SPLICS_S/L_-P2A^PO–ER^, and SPLICS_S/L_-P2A^PO–MT–ER^ were designed and obtained by custom gene synthesis (Thermo Fisher Scientific)^19,42^. Cytosolic and mitochondrially targeted aequorin constructs (cytAEQ and mtAEQmut) are described elsewere^43^. mCherryCAAX (mCherry-PM) (Plasmid #108886), ER marker ER-mScarlet (#85066), and Supernova plasmids (#85039 and # 85040) were purchased from Addgene. Supernova and SPLICS_S/L_ sequences were introduced into a plasmid backbone suitable for rAAV production.

### Cells culture

HeLa cells (ATCC) were grown in Dulbecco’s Modified Eagle’s Medium (DMEM) (Gibco; 41966-029) high glucose, 110 mg/L sodium pyruvate supplemented with 10% (vol/vol) Fetal Bovine Serum (FBS) (Gibco; Cat# 10270-106), 100 units per ml Penicillin and 100 μg/ml Streptomycin (Penicillin–Streptomycin solution 100X) (EuroClone; Cat# ECB3001D). Cells were maintained at 37 °C in a 5% CO_2_ atmosphere.

SOCE activation was induced 48 h after transfection by incubating the cells for 5 min at 37 °C in modified Krebs Ringer Buffer (KRB: 135 mM NaCl (Sigma-Aldrich; Cat# S9888), 5 mM KCl (Sigma-Aldrich; Cat# P3911), 0.4 mM KH_2_PO_4_ (Sigma-Aldrich; Cat# P0662), 1 mM MgSO_4_·(7 H_2_O) (Sigma-Aldrich; Cat# 63138), 1 mM MgCl_2_ (Sigma-Aldrich; Cat# M8266), 20 mM Hepes (Sigma-Aldrich; Cat# H3375), pH 7.4) supplemented with 20 µM 2,5-tertbutylhydroquinone (THBQ) (Sigma-Aldrich; Cat# 112976) or 100 nM thapsigargin (Sigma-Aldrich; Cat# T-9033), 100 μM histamine (Sigma-Aldrich; Cat# H7250), 100 or 200 μM EGTA (Sigma-Aldrich; Cat# E-4378). Then 2 mM CaCl_2_ (Sigma-Aldrich; Cat# 21115) was added to KRB and the cells incubated for 5 min at 37 °C. In the Ca^2+^ measurement experiments 2 mM CaCl_2_ was added where indicated in the figure. Untreated cells were kept in KRB supplemented with 2 mM CaCl_2_ for all the time of the treatment.

### Transfection

Twelve hours before transfection, HeLa cells were seeded at 60–80% confluence onto 13 mm diameter glass coverslips (for immunofluorescence contact sites analysis) or in 6-multiwell plates (for Western blotting analysis and aequorin measurements). Transfection was performed using the standard Ca^2+^ phosphate procedure^43^. Stock solutions are stored at −20 °C until the use: 2.5 M CaCl_2_ (Sigma-Aldrich; Cat# C-5080); HEPES Buffered Solution 2X (HBS 2X: 280 mM NaCl, 50 mM Hepes, 1.5 mM Na_2_HPO_4_·(7H_2_O) (Sigma-Aldrich; Cat# S9390), pH 7.12). Just before the transfection, the growth medium was replaced with fresh medium. For one 13 mm coverslip, 5 μl of 2.5 M CaCl_2_ was added to 4 μg of total DNA dissolved in Milli-Q H_2_O to reach a final volume of 50 μl. Routinely, for immunocytochemistry analysis 1 μg of SPLICS probes and 25 nM siRNAs (siRNA against human STIM1, SASI_Hs01_00107803 GGAUUUGACCCAUUCCGA[dT][dT], and siRNA against human Orai1, SASI_Hs01_00073221 CUGUGCACCUGUUUGCGCU[dT][dT]) were used to transfect one coverslip. The solution was then mixed under vortex with 50 μl of HBS 2X and incubated 30 min at room temperature. For Ca^2+^ measurements, cells were co-transfected with SPLICS probes and low-affinity mitochondrial aequorin (mtAeqmut) or cytosolic aequorin (cytAEQ). For one well of six multiwell plate, the amount of transfection mix was 15 μl of 2.5 M CaCl_2_, 12 μg of total DNA (2:1 ratio favouring pcDNA3.1(+) empty vector or SPLICS probes) in Milli-Q H_2_O to reach a final volume of 150 μl and 150 μl of HBS 2X. The transfection mix was then added directly to the cell monolayer, drop by drop. Ten hours after addition of the transfection mix, the medium was removed, and the cells were washed twice with Dulbecco’s Phosphate Buffered Saline (D-PBS) (EuroClone; Cat# ECB4004L) to remove excess of Ca^2+^ phosphate precipitates.

### Antibodies

The following reagents were used: monoclonal anti-KDEL (Abcam; ab176333), monoclonal anti-Tom20 (Santa Cruz Biotech., (F-10): Cat# sc-17764), monoclonal anti-PMP70 (Abcam; Cat# ab211533), monoclonal anti-STIM1 (BD Transduction Laboratories^TM^; Cat# 610954), polyclonal anti-Orai (Proscience; Cat# 30-571), monoclonal anti-β-actin (Sigma-Aldrich; Cat# A5441), mouse monoclonal anti-GFP (Santa Cruz Biotech.: Cat# sc-9996), polyclonal anti-β-tubulin (Cell Signaling; Cat# 2146c), AlexaFluor secondary antibody fluorophore-conjugated (Thermo Fisher: Goat anti-Rabbit IgG AlexaFluor 405, Cat# A-31556; Goat anti-Mouse IgG AlexaFluor 405, Cat#A-31553; Donkey anti-Rabbit IgG AlexaFluor 647, Cat# A-32795), secondary horseradish peroxidase-conjugated antibodies (Santa Cruz Biotech.; Goat anti-Rabbit IgG-HRP, Cat#sc-2004; Goat anti-Mouse IgG-HRP, Cat#sc-2005), 1:5000 in TBST.

### Western blotting analysis

HeLa cells were transfected in a six-well plate with siRNAs against human STIM1 (SASI_Hs01_00107803 GGAUUUGACCCAUUCCGA[dT][dT] or against human Orai1 (SASI_Hs01_00073221 CUGUGCACCUGUUUGCGCU [dT] [dT]). A scramble shRNA was used as control. Cells were recovered 48 hours after transfection cells were recovered from 6-multiwell plates and washed twice with PBS. Proteins were extracted by incubating the cells in ice-cold lysis buffer (50 mM Tris-HCl pH 7.4, 150 mM NaCl, 10 mM EGTA, 1% Triton X-100, 1 mM protease inhibitor cocktail (Sigma, Cat# P8340) for 20 min. Samples were then centrifuged at 16,000 × *g* for 10 min at 4 °C. Lysates were quantified by the Bradford assay (Bio-Rad, Cat# 500-0205), loaded on an 12% SDS/PAGE Tris-HCl polyacrylamide gel and blotted in Immobilon-PSQ PVDF Membrane (Merck Millipore, Cat# IPVH00010). The membrane was blocked for 1 h at room temperature using 5% non-fat dried milk (NFDM) in TBST (20 mM Tris-HCl, pH 7.4, 150 mM NaCl, 0.05% Tween-20) and incubated overnight with the specific primary antibody at 4 °C. Monoclonal anti-STIM1 1:1000 (BD Transduction Laboratories^TM^, Cat# 610954), polyclonal anti-ORAI1 1:1000 (Proscience, Cat# 30-571), and monoclonal anti-β-actin 1:30000 (Sigma-Aldrich; Cat# A5441) primary antibodies were used. Detection was carried out by incubation with secondary horseradish peroxidase-conjugated anti-rabbit or anti-mouse IgG antibodies for 1 h at room temperature followed by incubation with the chemiluminescent reagent LuminataClassico HRP substrate (Merck Millipore, Cat# WBLUO500).

### Immunocytochemistry

Forty-eight hours post transfection the cells, plated on 13-mm glass coverslips, were fixed for 20 min in a solution containing 3.7% (vol/vol) formaldehyde in D-PBS (Formaldehyde stock solution 37% in H_2_O (Sigma-Aldrich; Cat# F8775). The cells were then washed three times with D-PBS. Cell permeabilization was performed by incubation with 0.1% Triton^®^ X-100 *BioChemica* (PanReac AppliChem; Cat# A1388) in PBS for 20 min, followed by washing twice for 15 min in 1% Gelatine (Type B from bovine skin) (Sigma-Aldrich; Cat# G9382) in D-PBS at room temperature. The coverslips were then incubated for 90 min at 37 °C in a wet chamber with the specific primary antibody diluted 1:50 in D-PBS (monoclonal anti-KDEL, monoclonal anti-Tom20, monoclonal anti-PMP70. Staining was revealed by the incubation with specific AlexaFluor secondary antibody diluted 1:100 in D-PBS for 45 min at room temperature. After two washing steps of 15 min each, coverslips were mounted using Prolong^TM^ Diamond Antifade Mountant (Invitrogen; P36961). For Plasma Membrane labeling, the cells were transfected with PM -targeted mCherry vector (mCherry-CaaX(Hras) was a gift from Rob Parton (Addgene plasmid # 108886; http://n2t.net/addgene:108886; RRID:Addgene_108886^44^) and after fixing the coverslips mounted as above.

### SPLICs tracking in cultured cells

HeLa cells where seeded and transfected with SPLICS _S/L_-P2A^ER–MT^, then imaged on a custom widefield system based on a Zeiss Axiovert 100TV equipped with a Objective C-Apochromat 63x/1.20 W Corr M27 (n.a. 1.2) and a Retiga R3 (Teledyne QImaging). Z-stack were acquired with a voxel size of 90 nm in *xy* and 300 nm in *z*, every 10 s for 15 min. After acquisition Z-stack were digitally corrected using Huygens 3.3 3D deconvolution and an experimentally determined PSF. After deconvolution, dataset was corrected for photobleaching and 3D drifts using Fiji (https://imagej.net/Fiji). Corrected images were then segmented and tracked using three different softwares. For Fiji, analysis was performed using the trackmate plugin (https://imagej.net/TrackMate), segmentation was based on the Laplacian of gaussian algorithm, and tracking on the Linear Assignment Problem (LAP) algorithm. For Imaris (https://imaris.oxinst.com/), segmentation was based on isosurface reconstruction using the Ridler-Carval algorithm and tracking on LAP. For Icy (http://icy.bioimageanalysis.org/), segmentation was based on isosurface reconstruction using a K-mean algorithm and tracking on the Multiple Hypothesis Tracking algorithm. All tracks with a duration shorter than three points were excluded by the analysis.

### Confocal microscopy

Cells were observed with a Leica SP5-TCS-II-RS inverted confocal microscope upon illumination with laser at the wavelength of 405, 488, 514, and 543 nm using HCX Plan APO 63X/numerical aperture 1.40 oil-immersion objective. For all images, scanning mode was *xyz*, pinhole was set to 1 airy unit, format was 1024 × 1024, speed was 200 Hz, and pixel size was about 100 nm. To avoid crosstalk between fluorophores, sequential scans (scan-between frames) were performed and the beam splitter (dichroic) and the bandwidth were selected to make sure that the edges of the detection window are at least 10 nm apart from the excitation lines. Based on the number of scans between frames required the settings are: First Scan, activated laser 405 nm with intensity of around 40%, the dichroic is “Substrate” and the emission bandwidth of the detector is 420–470 nm; Second Scan: activated laser 488 nm with intensity of around 40%, the dichroic is “DD 488/543” and the emission bandwidth of the detector is 498–548 nm; Third Scan: activated laser 514 nm with intensity of around 40%, the dichroic is “DD 458/514” and the emission bandwidth of the detector is 524–574 nm; Fourth Scan: activated laser 514 nm (for excitation wavelength 588 of mCherrySync187) with intensity of around 40%, the dichroic is “488/543” and the emission bandwidth of the detector is 600–650 nm; Fifth Scan: activated laser 633 nm with intensity of around 40%, the dichroic is “TD 488/543/633” and the emission bandwidth of the detector is 643–693 nm. To count ER–mitochondria, ER–PM, PO–mitochondria, and PO–ER contacts a Z-stack was acquired for the whole depth of the cell by sampling at 290 nm in the *Z* plane. For in vivo imaging, cells were plated on 24 mm glass coverslips and transfected with empty and SPLICS_S/L_-P2A^ER–PM^ expressing vectors. Fluorescence was analyzed in living cells by Leica SP5-TCS-II-RS confocal microscope upon excitation at 514 nm wavelength and the single images were recorded. Confocal images were acquired with HCX Plan APO 63X/numerical aperture 1.40 oil-immersion objective (12 frames per second). Cells were maintained in KRB supplemented with 0.1% glucose and to induce ER Ca^2+^ depletion they were treated with 20 µM THBQ, 100 μM histamine, and 200 μM EGTA in KRB during acquisition of images.

### Image analysis

To count ER–mitochondria, ER–PM, PO–mitochondria, and PO–ER contacts, a complete Z-stack was processed using ImageJ (National Institutes of Health (NIH)): images were first convolved and the cells were selected using the freehand selection of ImageJ in the drawing/selection polygon tool and then processed using the “Quantification 1” plugin (https://github.com/titocali1/Quantification-Plugins). A 3D reconstruction of the resulting image was obtained using the Volume J plugin (https://github.com/titocali1/Quantification-Plugins). A selected face of the 3D rendering was then thresholded and used to count short and long contact sites through the “Quantification 2” plugin (https://github.com/titocali1/Quantification-Plugins).

### Ca^2+^ measurements

Ca^2+^ measurements were performed by co-transfecting HeLa cells in a six-well plate with cytAEQ or mtAEQmut with the SPLICS_S/L_-P2A^ER–PM^ and SPLICS_S/L_-P2A^ER–MT–PM^ moieties in a 1:3 ratio in favor of SPLICS plasmids. Twenty-four hours post transfection, cells were re-plated into a 96-well plate (Corning^®^; Cat# 3610). Aequorins was reconstituted by incubating cells for 90 min with 5 μM coelenterazine (Santa Cruz Biotech.; Cat# sc-205904) in KRB solution supplemented with 0.1% glucose and 1 mM CaCl_2_ at 37 °C. Luminescence measurements were carried out using a PerkinElmer EnVision plate reader equipped with two injector units. For mitochondrial Ca^2+^ measurements, after reconstitution, cells were placed in 70 μl of KRB solution with 0.1% glucose and 1 mM CaCl_2_ and luminescence from each well was measured for 30 s. During the experiment, 100 μM histamine at the final concentration was first injected to activate Ca^2+^ transients, and then a hypotonic, Ca^2+^-rich, digitonin (Sigma-Aldrich; Cat# D5628) containing solution was added to discharge the remaining aequorin pool. In cytosolic Ca^2+^ measurements, after reconstitution, cells were washed with KRB and then placed in 70 µl of KRB solution supplemented with 100 or 200 µM EGTA, 100 µM histamine and 100 nM thapsigargin or 20 µM THBQ, luminescence from each well was measured for 30 s. To activate Ca^2+^ transients, 2 mM CaCl_2_ were first injected and then a hypotonic, Ca^2+^-rich, digitonin-containing solution was added to discharge the remaining aequorin pool. Output data were analyzed and calibrated with a custom-made macro-enabled Excel workbook.

### Zebrafish husbandry and transgenic lines

All animal experiments were conducted on wild-type fish. Adult fish were maintained and raised in 5 l tanks with freshwater at 28 °C with a 12 h light/12 h dark cycle. Embryos were obtained from spontaneous spawnings and raised at 28 °C in Petri dishes containing fish water^45^. To perform experiments, both wt and s1102t:GAL4 fish were used. All experiments were conducted on 24 hours post fertilization (hpf) embryos.

### Zebrafish imaging

The pT2-DsRed-UAS-SPLICS_S_-P2A vector has been already described^19^. Before injections, all plasmids were diluted in Danieau solution (58 mM NaCl, 0.7 mM KCl, 0.4 mM MgSO_4_, 0.6 mM Ca(NO_3_)_2_, 5 mM HEPES pH 7.6) and 0.5% phenol red. At 24 hpf, embryos were screened for fluorescence, dechorionated, and anesthetised with tricaine. They were then mounted on 35 × 10 mm glass bottom Petri dishes (Ted Pella, INC. Prod. No 14023-20) in low melting agarose (1.3%, EuroClone). Fish water containing tricaine methanesulfonate 0.61 mM (Sigma) was added in the Petri dishes, in order to keep fish anesthetised. Mounted fish were imaged at RT (20–23 °C) using a Leica TSC SP5 inverted confocal microscope, using a HCX PL APO 63X/numerical aperture 1.40-0.60 oil-immersion objective. To image ER–mitochondria contacts, a Z-stack of the cell was acquired. Representative time-lapse recordings were acquired with a frame interval of 12–18 s for a total time of 10 min. Time-lapse movies were analyzed with Fiji in order to obtain kymographs.

### Statistical analysis, graphics, and figure assembly

Data are given as means ± SEM or SD as specified in the figure legends. Where two groups were compared and followed a Gaussian distribution, statistical significance was calculated by unpaired Student’s two-tailed *t* test, otherwise Mann–Whitney test was used. All statistical significance was calculated at *p* ≤ 0.05, using GraphPad Prism 8 (GraphPad, San Diego, CA, USA). For all the analysis, the samples were collected and processed simultaneously and, therefore, no randomization was appropriate. *n* = number of at least three independent experiments or cells from at least three independent transfections. The exact values of *n* and their means are indicated in the figure legends. *p ≤ 0.05, **p ≤ 0.01 ***p ≤ 0.001 ****p ≤ 0.0001. Data obtained by tracking analysis do not respect normality assumption and were then analyzed by Mann–Whitney or Kolmgorov–Smirnov tests, previous outliers’ removal. We have complied with all relevant ethical regulations. All the protocols were approved by the University of Padova and Italian Ministry of Education and Research for Danio Rerio and by the University of Heidelberg for Sprague-Dawley rats.

### Reporting Summary

Further information on research design is available in the Nature Research Reporting Summary linked to this article.

## Supplementary information

Supplementary Information

Supplementary Movie 1

Supplementary Movie 2

Supplementary Movie 3

Supplementary Movie 4

Supplementary Movie 5

Supplementary Movie 6

Reporting Summary

Description of Additional Supplementary Files

## Data Availability

The data that support this study are present in the manuscript and supplementary information, and are available from the corresponding author upon request. Source data are provided with this paper.

## Source data

Source Data

## Code availability

Quantification Plugins are available at https://github.com/titocali1/Quantification-Plugins.

## References

[1] Phillips MJ, Voeltz GK (2016). Structure and function of ER membrane contact sites with other organelles. Nat. Rev. Mol. Cell Biol..

[2] Scorrano L (2019). Coming together to define membrane contact sites. Nat. Commun..

[3] Rusinol AE, Cui Z, Chen MH, Vance JE (1994). A unique mitochondria-associated membrane fraction from rat liver has a high capacity for lipid synthesis and contains pre-Golgi secretory proteins including nascent lipoproteins. J. Biol. Chem..

[4] Wieckowski MR, Giorgi C, Lebiedzinska M, Duszynski J, Pinton P (2009). Isolation of mitochondria-associated membranes and mitochondria from animal tissues and cells. Nat. Protoc..

[5] Mannella CA, Buttle K, Rath BK, Marko M (1998). Electron microscopic tomography of rat-liver mitochondria and their interaction with the endoplasmic reticulum. Biofactors.

[6] Csordas G (2006). Structural and functional features and significance of the physical linkage between ER and mitochondria. J. Cell Biol..

[7] de Brito OM, Scorrano L (2008). Mitofusin 2 tethers endoplasmic reticulum to mitochondria. Nature.

[8] Murley A (2013). ER-associated mitochondrial division links the distribution of mitochondria and mitochondrial DNA in yeast. Elife.

[9] Friedman JR (2011). ER tubules mark sites of mitochondrial division. Science.

[10] Wu Y (2017). Contacts between the endoplasmic reticulum and other membranes in neurons. Proc. Natl Acad. Sci. USA.

[11] Elgass KD, Smith EA, LeGros MA, Larabell CA, Ryan MT (2015). Analysis of ER-mitochondria contacts using correlative fluorescence microscopy and soft X-ray tomography of mammalian cells. J. Cell Sci..

[12] Filadi R (2015). Mitofusin 2 ablation increases endoplasmic reticulum-mitochondria coupling. Proc. Natl Acad. Sci. USA.

[13] Rizzuto R, Brini M, Murgia M, Pozzan T (1993). Microdomains with high Ca2+ close to IP3-sensitive channels that are sensed by neighboring mitochondria. Science.

[14] Brunstein M, Wicker K, Herault K, Heintzmann R, Oheim M (2013). Full-field dual-color 100-nm super-resolution imaging reveals organization and dynamics of mitochondrial and ER networks. Opt. Express.

[15] Bottanelli F (2016). Two-colour live-cell nanoscale imaging of intracellular targets. Nat. Commun..

[16] Tubbs, E. & Rieusset, J. Study of endoplasmic reticulum and mitochondria interactions by in situ proximity ligation assay in fixed cells. *J. Vis. Exp.*10.3791/54899 (2016). 10.3791/54899 PMC5226377 28060261

[17] Csordas G (2010). Imaging interorganelle contacts and local calcium dynamics at the ER-mitochondrial interface. Mol. Cell.

[18] Shi F (2018). Optogenetic control of endoplasmic reticulum-mitochondria tethering. ACS Synth. Biol..

[19] Cieri D (2018). SPLICS: a split green fluorescent protein-based contact site sensor for narrow and wide heterotypic organelle juxtaposition. Cell Death Differ..

[20] Yang, Z., Zhao, X., Xu, J., Shang, W. & Tong, C. A novel fluorescent reporter detects plastic remodeling of mitochondria-ER contact sites. *J. Cell Sci.*10.1242/jcs.208686 (2018). 10.1242/jcs.208686 29158224

[21] Harmon M, Larkman P, Hardingham G, Jackson M, Skehel P (2017). A bi-fluorescence complementation system to detect associations between the endoplasmic reticulum and mitochondria. Sci. Rep..

[22] Kakimoto Y (2018). Visualizing multiple inter-organelle contact sites using the organelle-targeted split-GFP system. Sci. Rep..

[23] Shai N (2018). Systematic mapping of contact sites reveals tethers and a function for the peroxisome-mitochondria contact. Nat. Commun..

[24] He L (2017). Optical control of membrane tethering and interorganellar communication at nanoscales. Chem. Sci..

[25] Macpherson LJ (2015). Dynamic labelling of neural connections in multiple colours by trans-synaptic fluorescence complementation. Nat. Commun..

[26] Kim JH (2011). High cleavage efficiency of a 2A peptide derived from porcine teschovirus-1 in human cell lines, zebrafish and mice. PloS One.

[27] Bravo-Sagua R (2016). mTORC1 inhibitor rapamycin and ER stressor tunicamycin induce differential patterns of ER-mitochondria coupling. Sci. Rep..

[28] Yagita Y (2017). Deficiency of a retinal dystrophy protein, Acyl-CoA binding domain-containing 5 (ACBD5), impairs peroxisomal beta-oxidation of very-long-chain fatty acids. J. Biol. Chem..

[29] Soukupova M, Sprenger C, Gorgas K, Kunau WH, Dodt G (1999). Identification and characterization of the human peroxin PEX3. Eur. J. Cell Biol..

[30] Hoepfner D, Schildknegt D, Braakman I, Philippsen P, Tabak HF (2005). Contribution of the endoplasmic reticulum to peroxisome formation. Cell.

[31] Sugiura A, Mattie S, Prudent J, McBride HM (2017). Newly born peroxisomes are a hybrid of mitochondrial and ER-derived pre-peroxisomes. Nature.

[32] Besprozvannaya, M. *et al*. GRAM domain proteins specialize functionally distinct ER-PM contact sites in human cells. *Elife*10.7554/eLife.31019 (2018). 10.7554/eLife.31019 PMC5823543 29469807

[33] Valm AM (2017). Applying systems-level spectral imaging and analysis to reveal the organelle interactome. Nature.

[34] Luo W (2016). Supernova: a versatile vector system for single-cell labeling and gene function studies in vivo. Sci. Rep..

[35] Henne M (2019). And three’s a party: lysosomes, lipid droplets, and the ER in lipid trafficking and cell homeostasis. Curr. Opin. Cell Biol..

[36] Murley A, Nunnari J (2016). The emerging network of mitochondria-organelle contacts. Mol. Cell.

[37] Wu, H., Carvalho, P. & Voeltz, G. K. Here, there, and everywhere: the importance of ER membrane contact sites. *Science*10.1126/science.aan5835 (2018). 10.1126/science.aan5835 PMC6568312 30072511

[38] Bading H, Greenberg ME (1991). Stimulation of protein tyrosine phosphorylation by NMDA receptor activation. Science.

[39] Zhang SJ (2011). A signaling cascade of nuclear calcium-CREB-ATF3 activated by synaptic NMDA receptors defines a gene repression module that protects against extrasynaptic NMDA receptor-induced neuronal cell death and ischemic brain damage. J. Neurosci..

[40] Wiegert JS, Bengtson CP, Bading H (2007). Diffusion and not active transport underlies and limits ERK1/2 synapse-to-nucleus signaling in hippocampal neurons. J. Biol. Chem..

[41] Zhang SJ (2007). Decoding NMDA receptor signaling: identification of genomic programs specifying neuronal survival and death. Neuron.

[42] Gonzalo S, Greentree WK, Linder ME (1999). SNAP-25 is targeted to the plasma membrane through a novel membrane-binding domain. J. Biol. Chem..

[43] Ottolini D, Cali T, Brini M (2014). Methods to measure intracellular Ca(2+) fluxes with organelle-targeted aequorin-based probes. Methods Enzymol..

[44] Ariotti N (2018). Ultrastructural localisation of protein interactions using conditionally stable nanobodies. PLoS Biol..

[45] Bergamin G, Cieri D, Vazza G, Argenton F, Mostacciuolo ML (2016). Zebrafish Tg(hb9:MTS-Kaede): a new in vivo tool for studying the axonal movement of mitochondria. Biochim Biophys. Acta.

