# Supplementary material for: An expanded palette of improved SPLICS reporters detects multiple organelle contacts in vitro and in vivo
Source: Nat Commun. 2020 Nov 27;11:6069. doi: 10.1038/s41467-020-19892-6 (PMC7699637; doi:10.1038/s41467-020-19892-6)
Supplement: Supplementary file 1 — Supplementary Information [file 41467_2020_19892_MOESM1_ESM.docx]

**Supplementary Information**

**An expanded palette of improved SPLICS reporters detects multiple organelle contacts in vitro and in vivo**

Francesca Vallese^1,#^, Cristina Catoni^2,#^, Domenico Cieri^1^, Lucia Barazzuol^1^, Omar Ramirez^3^, Valentina Calore^1^, Massimo Bonora^4,5^, Flavia Giamogante^1^, Paolo Pinton^4,5^, Marisa Brini^2,*^ and Tito Calì^1,6,*^

^1^ Department of Biomedical Sciences, University of Padova, Padova, Italy. ^2^ Department of Biology, University of Padova, Padova, Italy. ^3^ Department of Neurobiology, Interdisciplinary Center for Neurosciences, Heidelberg University, Heidelberg, Germany. ^4^ Department of Morphology, Surgery and Experimental Medicine, Section of General Pathology, University of Ferrara, Ferrara, Italy. ^5^ Laboratory for Technologies of Advanced Therapies (LTTA), University of Ferrara, Ferrara, Italy. ^6^ Padova Neuroscience Center (PNC), University of Padova, Padova, Italy. ^7^ These authors contributed equally: Vallese, F; Catoni, C. *e-mail: [tito.cali@unipd.it](mailto:tito.cali@unipd.it) or [marisa.brini@unipd.it](mailto:marisa.brini@unipd.it) .

**
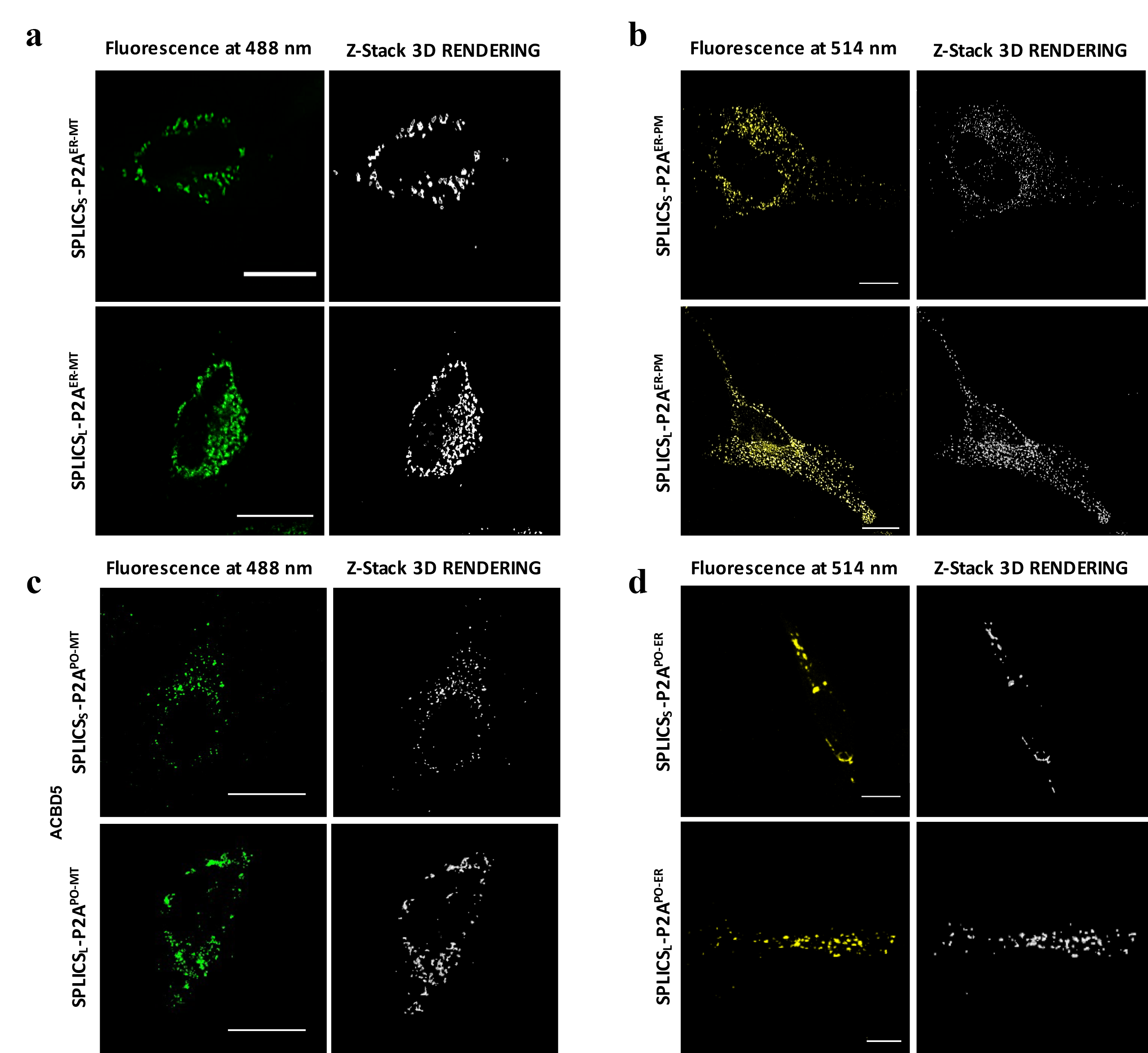
**

**Supplementary Figure 1**

Representative confocal pictures and 3D rendered signal derived from integral Z-stack analysis of HeLa cells expressing the SPLICS_S_-P2A (upper panels) or SPLICS_L_-P2A (bottom panels) probe. SPLICS-P2A^ER-MT^ (**A**), SPLICS-P2A^PO-MT^ (**B**), SPLICS-P2A^ER-PM^ (**C**) and SPLICS-P2A^PO-ER^ (**D**). Quantification of 3D rendering can reveal statistically significant differences between short and long range membrane contact sites as shown in Figure 1 of the main text. Scale bar 10 μM

**
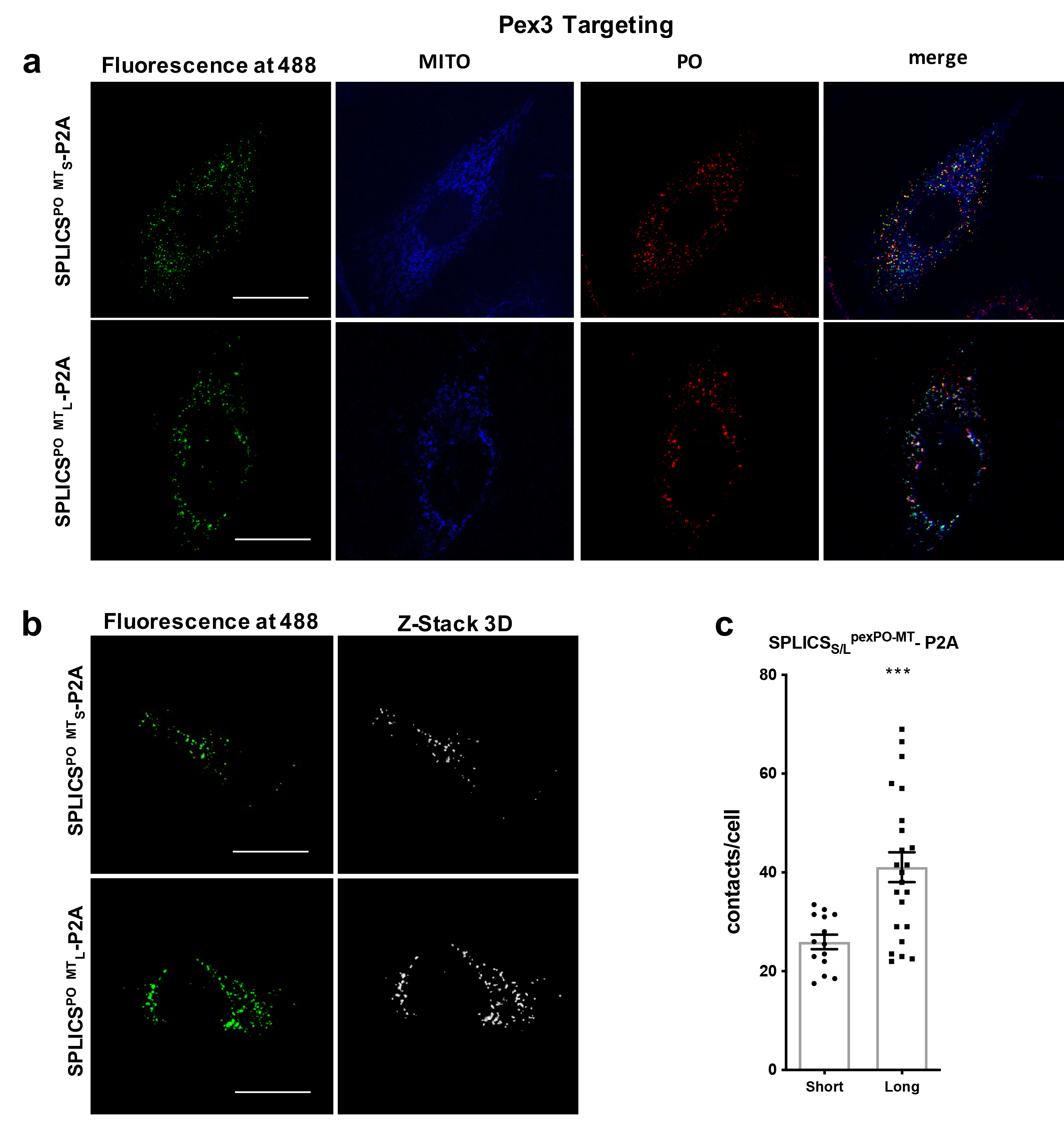
Supplementary Figure 2**

SPLICS_S/L_ ^PO-MT^ reporter with a PO targeting sequence from the peroxisomal protein PEX3. These SPLICS_S/L_-P2A^PO-MT^ PEX3 probes have a comparable efficiency. (**a)** Colocalization of SPLICS_S/L_ (488 nm) with mitochondria (mtHSP60) and PO (PMP70). (**b)** 3D rendered signal derived from integral Z-stack analysis of HeLa cells expressing the SPLICS_S_-P2A^PO-MT^ PEX3 probe (upper panels) or SPLICS_L_-P2A^PO-MT^ PEX3 probe (bottom panels). **(c)** Quantification of SPLICS_S/L_-P2A^PO-MT^ PEX3 contacts by 3D rendering of complete z-stacks. Mean ± SEM: SPLICS_S_-P2A^PO-MT^ 25.93 ± 1.47, n=14 cells; SPLICS_L_-P2A^PO-MT^ 41.07 ± 3.02, n=23 cells. *** P ≤ 0.001 unpaired two-tailed t-test. Scale bar 10 μM.


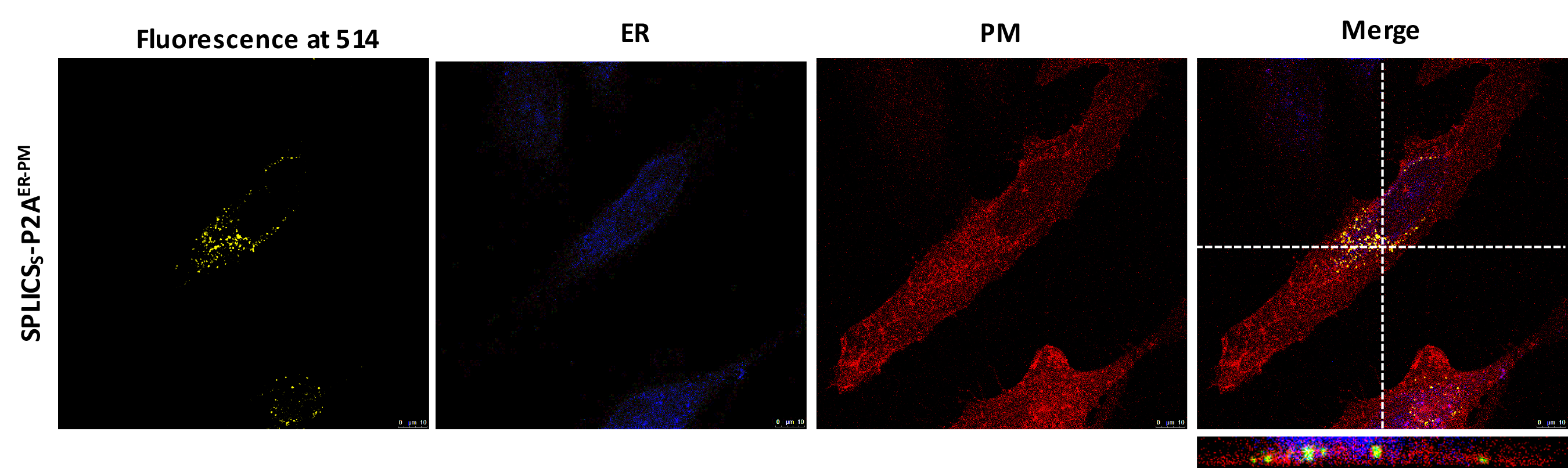


**Supplementary Figure 3**

HeLa cells co-expressing the SPLICS_S_-P2A^ER-PM^ along with a PM-targeted mCherry (CAAX-mCherry). Immunofluorescence with an anti KDEL antibody is shown in blue to detect the ER. The sagittal section over a whole Z-stack shows the specific presence of contact sites between the ER and the PM below the cell surface. Scale bar is 10μm.

**
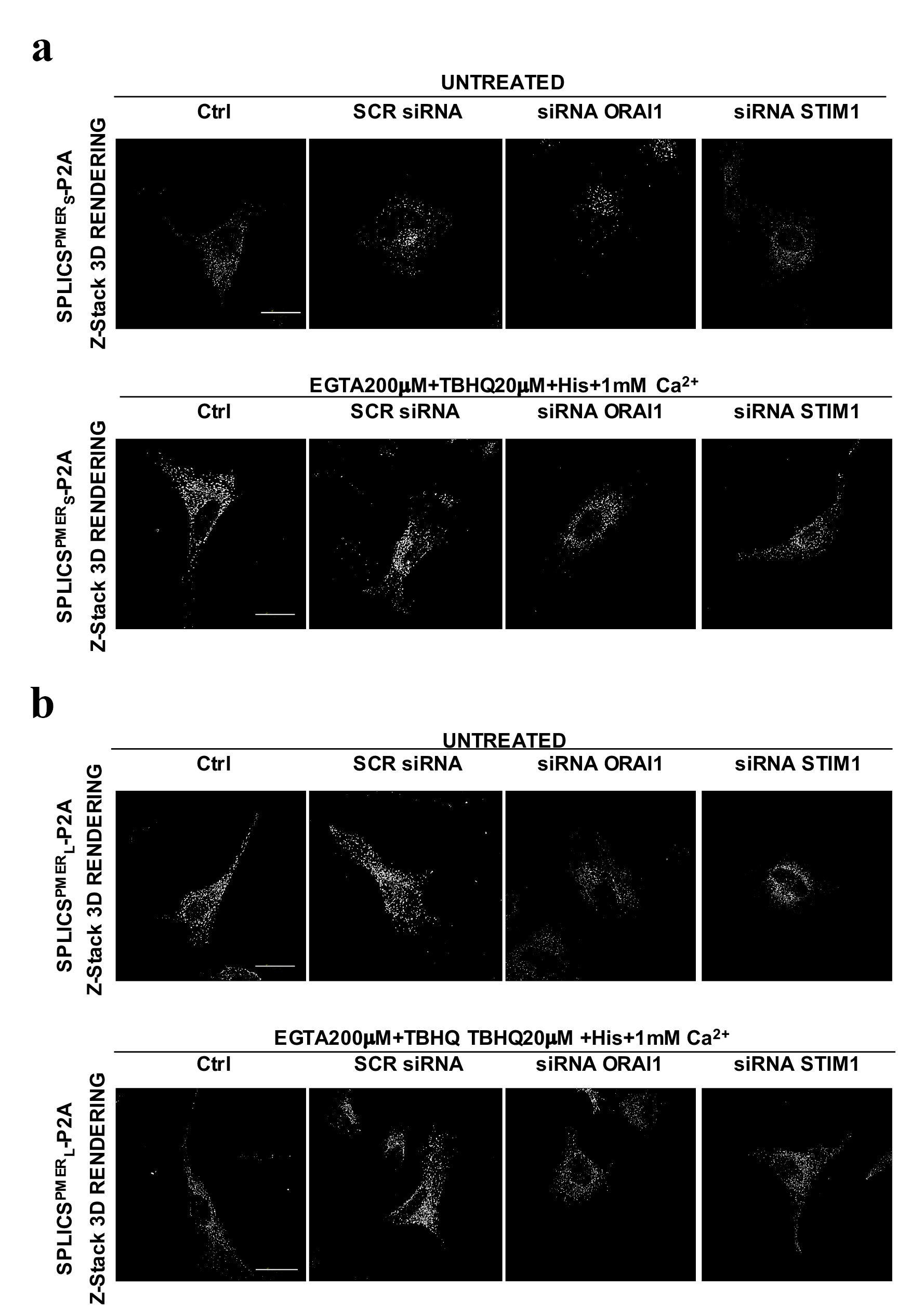
**

**Supplementary Figure 4**

Effect of STIM1/ORAI1 silencing and ER Ca^2+^ depletion on short (**A**) and long (**B**) range contacts between ER and PM. 3D reconstructions from Z-stack images of HeLa cells expressing the SPLICS_S/L_-P2A^ER-PM^ untreated (top) or treated for 5 min (bottom) with 200μM EGTA, 20µM THBQ, 100μM histamine and then for additional 5 min with 1mM CaCl_2_ supplemented to KRB. Scale bar 25µm. Related to Figure 3**.**

**
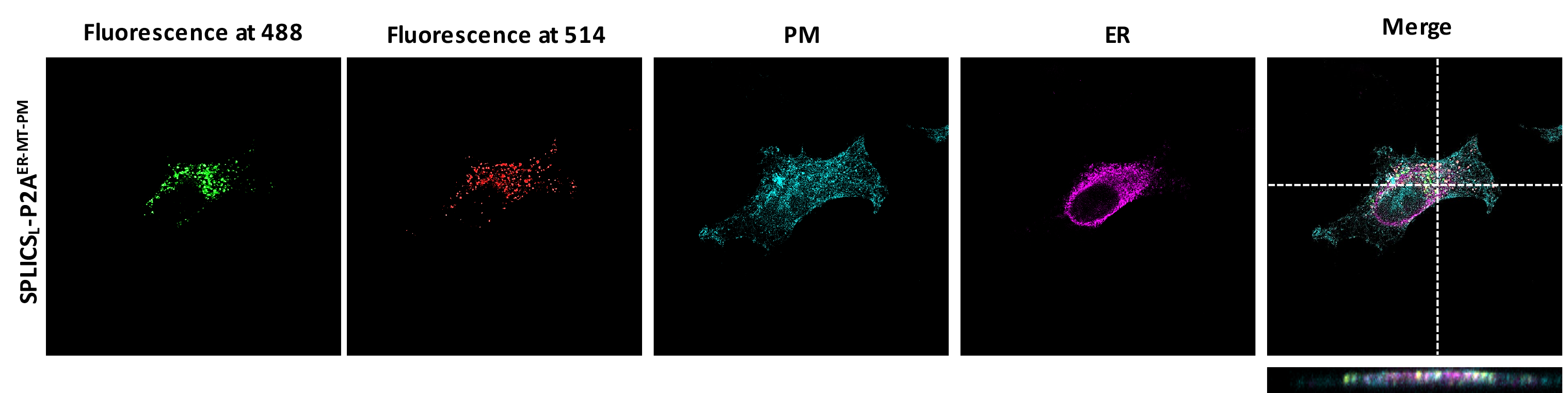
**

**Supplementary Figure 5**

HeLa cells co-expressing the SPLICS_L_-P2A^ER-MT-PM^ along with a PM-targeted mCherry (CAAX-mCherry). Complemented YFP1-10 (fluorescence at 514 nm) and CAAX-mCherry (PM) are shown in pseudo colours, red and cyan, respectively. Immunofluorescence with an anti KDEL antibody is shown in magenta to detect the ER. The sagittal section over a whole Z-stack shows the specific presence of contact sites at the given cell boundaries. Scale bar is 10μm.

**
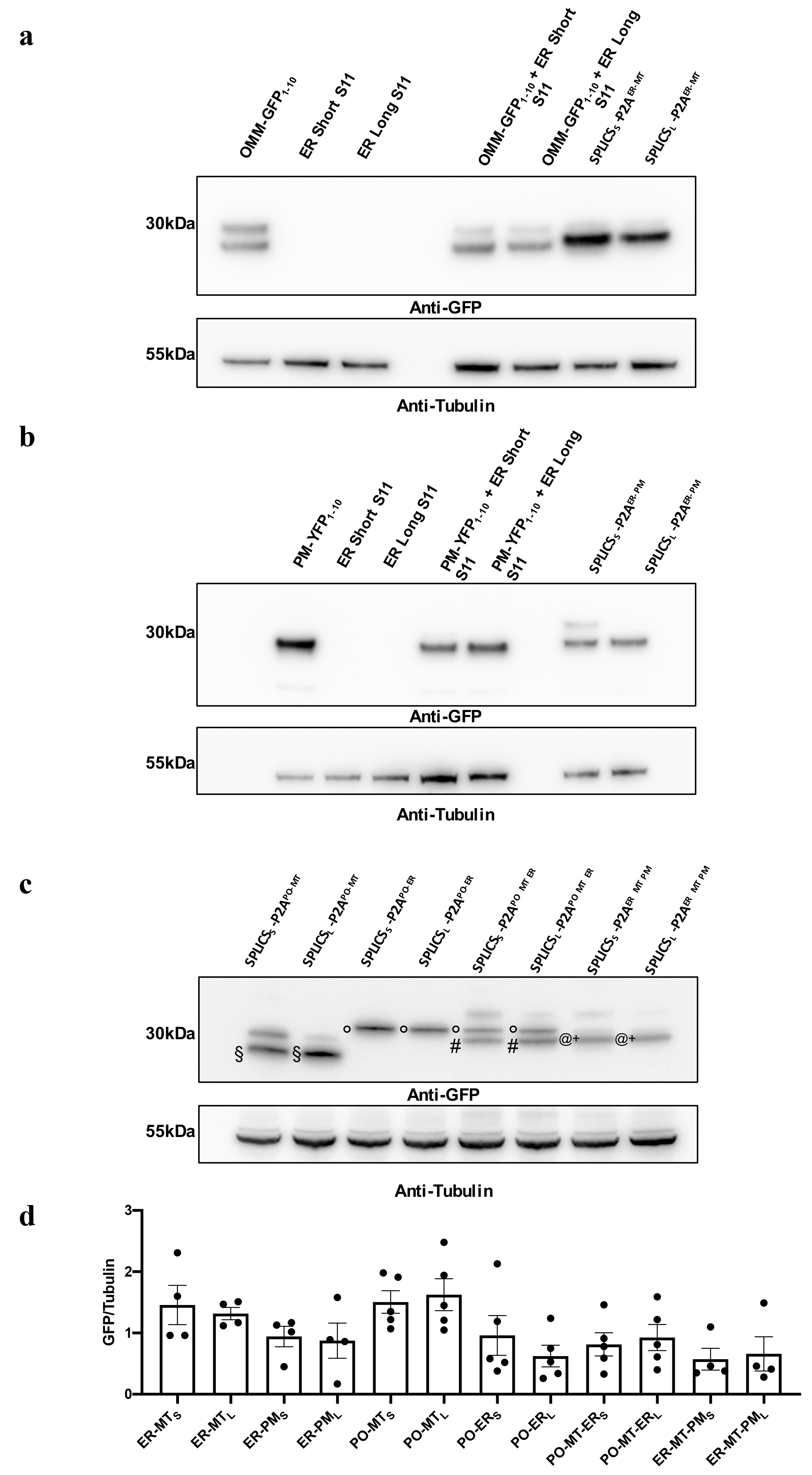
**

**Supplementary Figure 6**

Western blot analysis and quantification of lysates of HeLa cells transfected with the indicated constructs a) OMMGFP1-10, ERShort S11 and ERLong S11, b) PMYFP1-10, ERShort S11 and ERLong S11 and c) SPLICS-P2A sensors and probed with an anti-GFP antibody. The membrane is probed with an anti-Tubulin antibody to control equal loading. d) quantification of the indicated SPLICS constructs. ^§^OMM-GFP1-10: 27.3kDa; °ER-YFP: 32.7kDa; ^#^OMM-GFP1-10: 30.6kDa; ^@^OMM-GFP1-10: 31kDa; ^+^PM-YFP1-10: 30.5kDa. The OMM-GFP1-10 constructs always appears as two bands being the upper one the unprocessed pre-import form. Data mean±SEM were obtained from 4-5 independent western blottings.

**Supplementary Figure 7**

Aminoacidic sequences of the SPLICS sensors used in this study and relative expected molecular weights of the organelle targeted fragments.

**
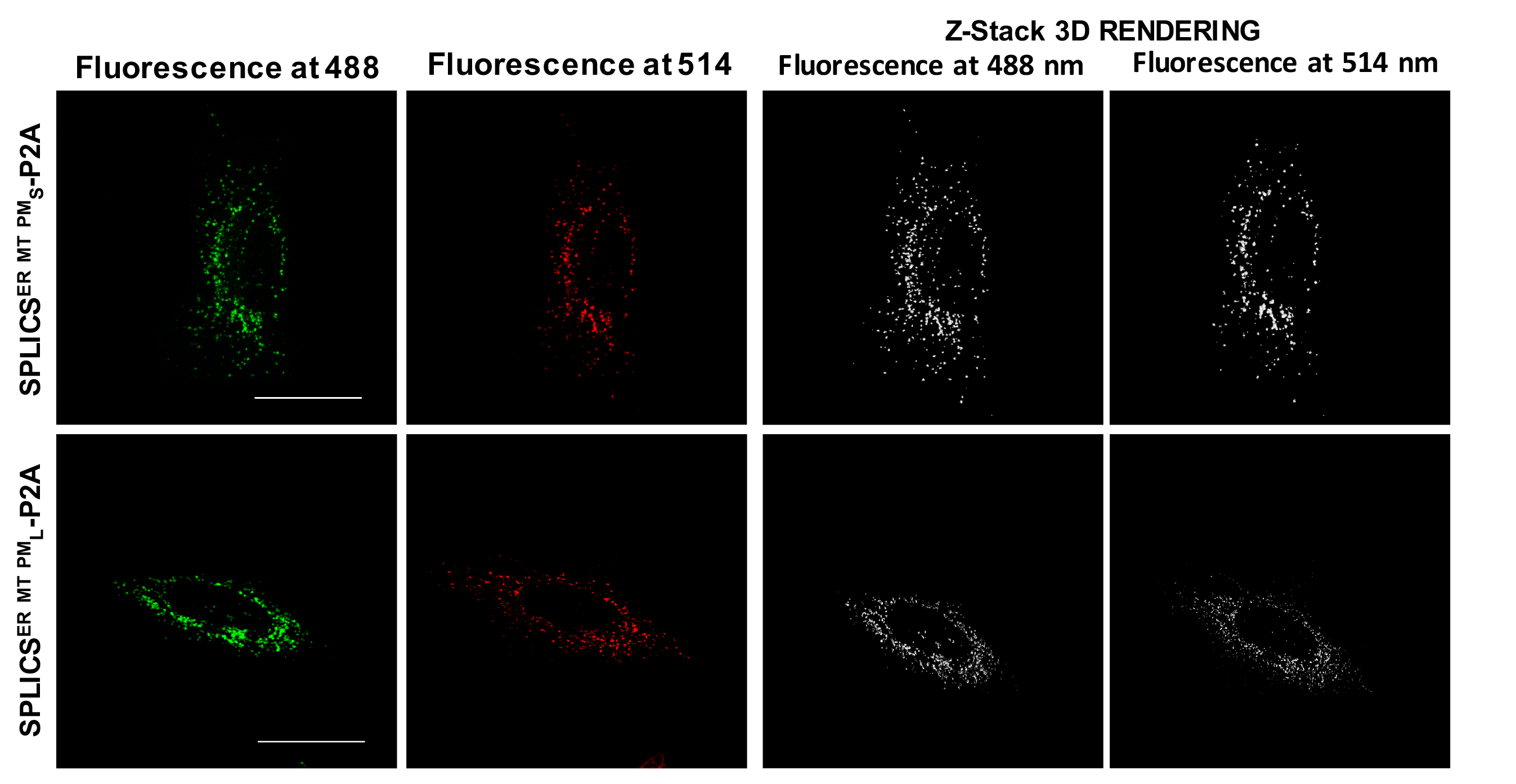
**

**Supplementary Figure 8**

Representative confocal pictures and 3D rendered signal derived from integral Z-stack analysis of HeLa cells expressing SPLICS-P2A^ER-MT-PM^ either short (upper panels) or long (bottom panels). Excitation at 488nm shows the ER-MT while excitation at 514nm (in red pseudocolor) shows the ER-PM contact sites. Scale bar 10 μM.

**
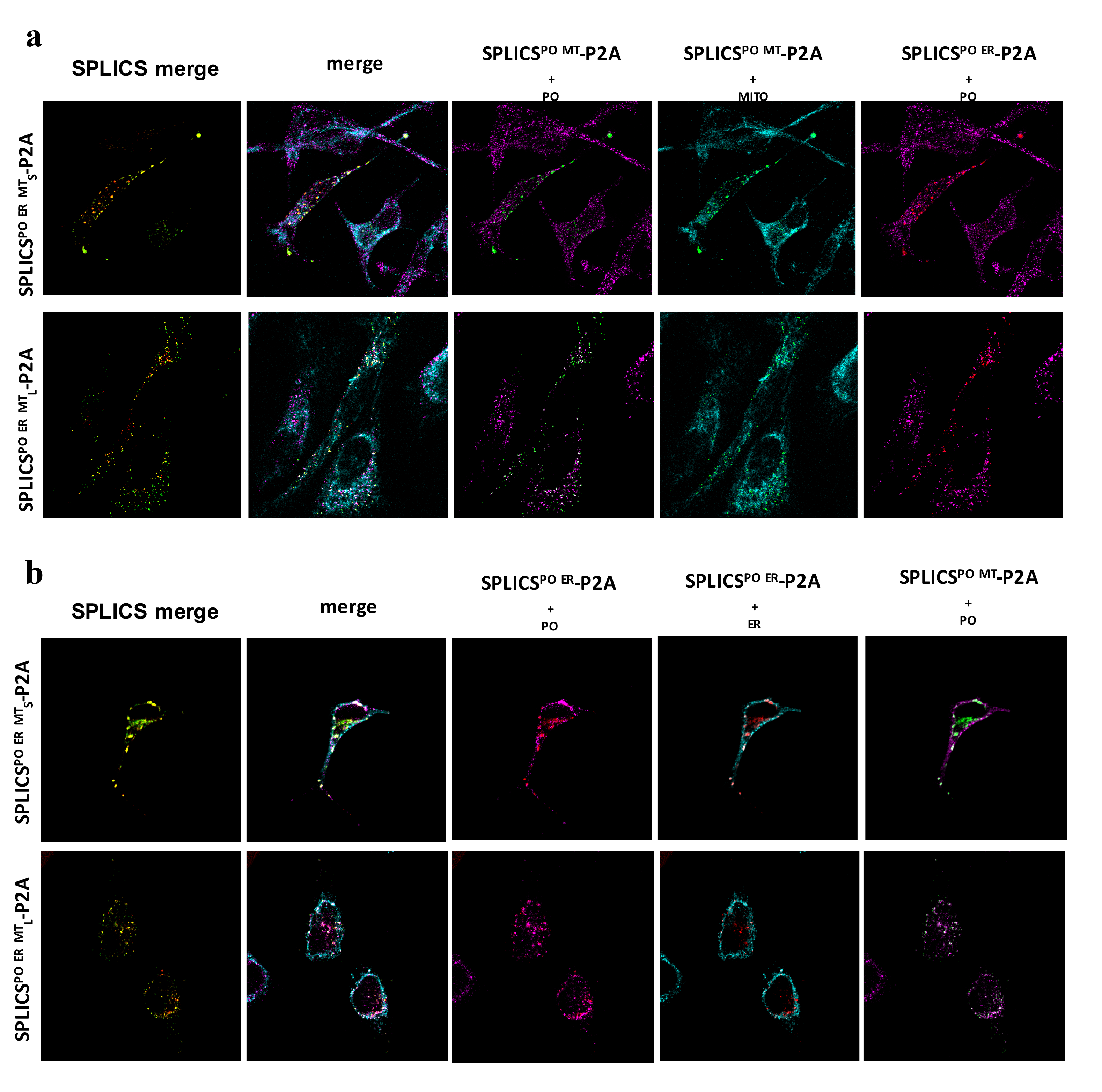
**

**Supplementary Figure 9**

HeLa cells co-expressing the SPLICS_S/L_-P2A^PO-ER-MT^, shown are the merge panels relative to Figure 5 C and E. Scale bar 10 μM.

**
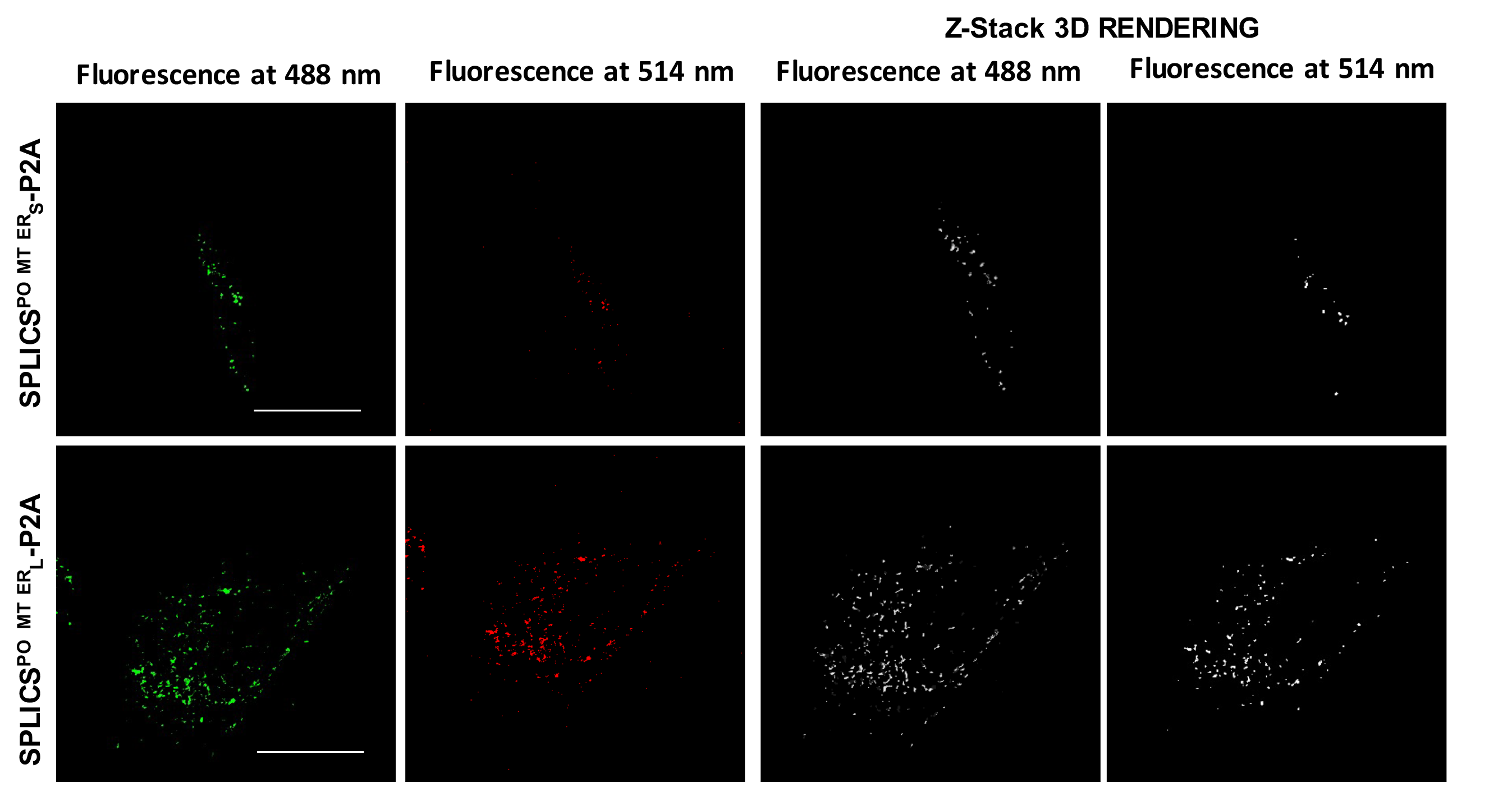
**

**Supplementary Figure 10**

Representative confocal pictures and 3D rendered signal derived from integral Z-stack analysis of HeLa cells expressing SPLICS-P2A^PO-MT-PM^ either short (upper panels) or long (bottom panels). Excitation at 488 nm shows the PO-MT while excitation at 514 nm (in red pseudocolor) shows the PO-ER contact sites. Scale bar 10 μM.

**
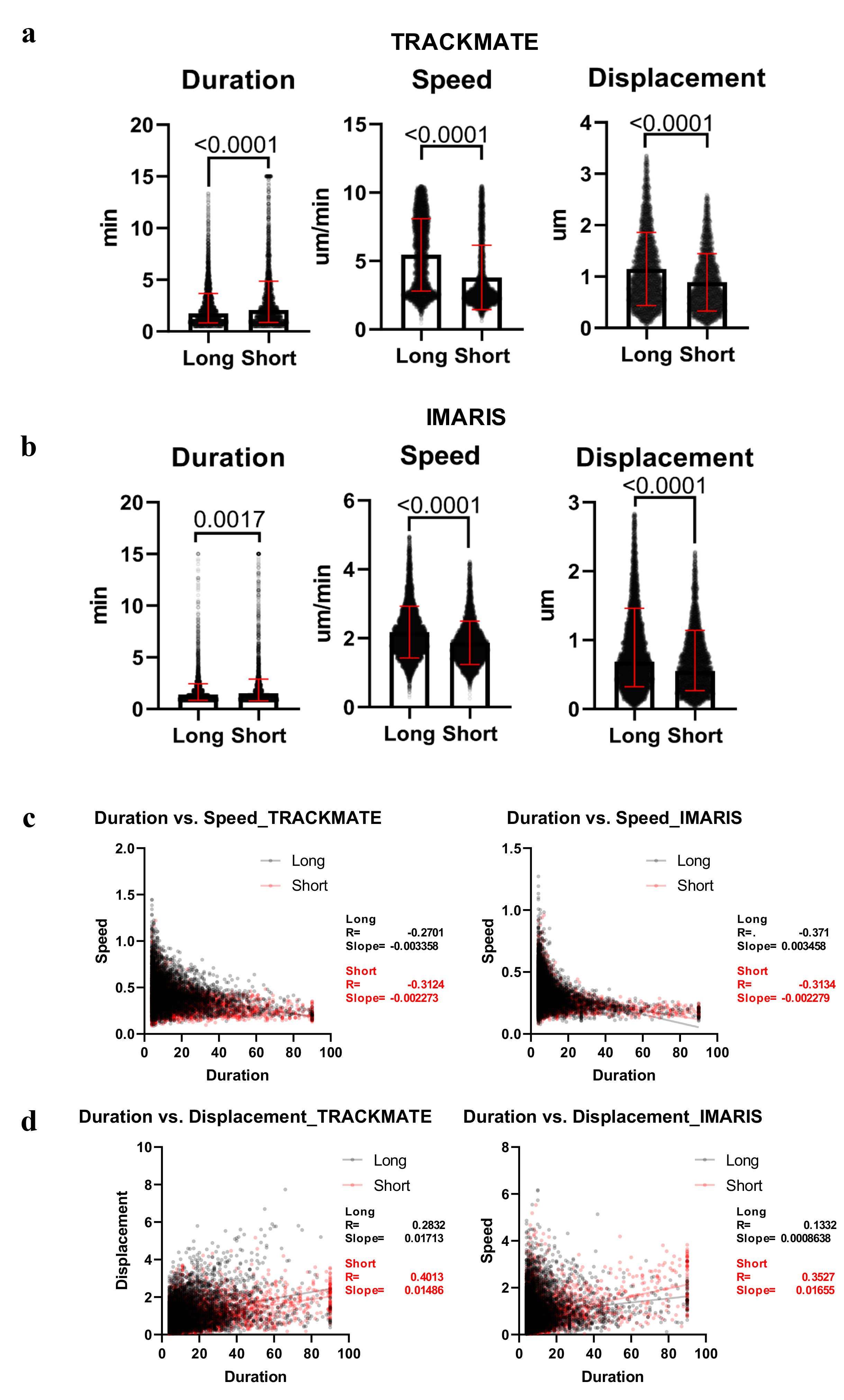
**

**Supplementary Figure 11**

SPLICS tracking by alternative algorithms. (**a**), Tracks quantitative analysis obtained by the Trackmate method of track duration (left panel, geometric mean  ± geometric SD: ER-MT_S_ 2.067 ± 2.349, *n* = 4154 tracks from 6 cells; ER-MT_L_ 1.753 ± 2.100, *n* = 5287 tracks form 6 cells), track speed (middle panel, geometric mean ± geometric SD: ER-MT_S_ 3.232 ± 1.730, *n* = 4154 tracks from 6 cells; ER-MT_L_ 4.749 ± 1.735, *n* = 5310 tracks form 6 cells), track displacement (right panel, geometric mean ± geometric SD: ER-MT_S_ 0.7125 ± 2.040, *n* = 4052 tracks from 6 cells; ER-MT_L_ 0.9056 ± 2.035, *n* = 5088 tracks form 6 cells). (**b**), Tracks quantitative analysis obtained by the Imaris method of track duration (left panel, geometric mean ± geometric SD: ER-MT_S_ 1.518 ± 1.908, *n* = 4142 tracks from 6 cells; ER-MT_L_ 1.422 ± 1.720, *n* = 4484 tracks form 6 cells), track speed (middle panel, geometric mean ± geometric SD: ER-MT_S_ 1.769 ± 1.392, *n* = 7132 tracks from 6 cells; ER-MT_L_ 2.054 ± 1.409, *n* = 8890 tracks form 6 cells), track displacement (right panel, geometric mean ± geometric SD: ER-MT_S_ 0.5519 ± 2.067, *n* = 6475 tracks from 6 cells; ER-MT_L_ 0.6888 ± 2.122, *n* = 8759 tracks form 6 cells). (**c**) Duration/speed and (**d**) Duration/Displacement analysis performed with Trackmate and Imaris relative to the experiments performed in (**a**) and (**b**).
