# Supplementary material for: An expanded palette of improved SPLICS reporters detects multiple organelle contacts in vitro and in vivo
Source: Nat Commun. 2020 Nov 27;11:6069. doi: 10.1038/s41467-020-19892-6 (PMC7699637; doi:10.1038/s41467-020-19892-6)
Supplement: Supplementary file 8 — Reporting Summary [file 41467_2020_19892_MOESM8_ESM.pdf]

nature research

Corresponding author(s): Marisa Brini
Tito Calì

Last updated by author(s): 27/10/2020

Reporting Summary

Nature Research wishes to improve the reproducibility of the work that we publish. This form provides structure for consistency and transparency in reporting. For further information on Nature Research policies, see Authors & Referees and the Editorial Policy Checklist .

Statistics

For all statistical analyses, confirm that the following items are present in the figure legend, table legend, main text, or Methods section.

n/a	Confirmed	
[ ]	[x]	The exact sample size (*n*) for each experimental group/condition, given as a discrete number and unit of measurement
[ ]	[x]	A statement on whether measurements were taken from distinct samples or whether the same sample was measured repeatedly
[ ]	[x]	The statistical test(s) used AND whether they are one- or two-sided<br>*Only common tests should be described solely by name; describe more complex techniques in the Methods section.*
[x]	[ ]	A description of all covariates tested
[ ]	[x]	A description of any assumptions or corrections, such as tests of normality and adjustment for multiple comparisons
[ ]	[x]	A full description of the statistical parameters including central tendency (e.g. means) or other basic estimates (e.g. regression coefficient) AND variation (e.g. standard deviation) or associated estimates of uncertainty (e.g. confidence intervals)
[ ]	[x]	For null hypothesis testing, the test statistic (e.g. *F*, *t*, *r*) with confidence intervals, effect sizes, degrees of freedom and *P* value noted<br>*Give P values as exact values whenever suitable.*
[x]	[ ]	For Bayesian analysis, information on the choice of priors and Markov chain Monte Carlo settings
[x]	[ ]	For hierarchical and complex designs, identification of the appropriate level for tests and full reporting of outcomes
[x]	[ ]	Estimates of effect sizes (e.g. Cohen's *d*, Pearson's *r*), indicating how they were calculated

Our web collection on statistics for biologists contains articles on many of the points above.

Software and code

Policy information about availability of computer code

Data collection	Data was acquired using software supplied by instrument manufacturer (ZEISS). Microsoft office 365, ImageJ (FIJI) V.2.0.0-rc-69/1.52p, Imaris 9.6, Icy JDK 8, MacOS Catalina, Prism8
Data analysis	Prism 8

For manuscripts utilizing custom algorithms or software that are central to the research but not yet described in published literature, software must be made available to editors/reviewers. We strongly encourage code deposition in a community repository (e.g. GitHub). See the Nature Research guidelines for submitting code & software for further information.

Data

Policy information about availability of data

All manuscripts must include a data availability statement. This statement should provide the following information, where applicable:
- Accession codes, unique identifiers, or web links for publicly available datasets
- A list of figures that have associated raw data
- A description of any restrictions on data availability

Source data are provided with this paper

Field-specific reporting

Please select the one below that is the best fit for your research. If you are not sure, read the appropriate sections before making your selection.

[x] Life sciences [ ] Behavioural & social sciences [ ] Ecological, evolutionary & environmental sciences

Life sciences study design

All studies must disclose on these points even when the disclosure is negative.

Sample size	Sample sizes for all statistical evaluations are indicated in Figure captions and were determined as reported in Cieri et al, Cell Death Differ, 2018
Data exclusions	No raw image data was excluded, image processing steps are detailed in the on-line methods.
Replication	All attempts were succesfull, the number of repeated imaging experiments with similar outcomes are indicated in the figure captions
Randomization	Random Images are acquired and quantified through an automated plugin (Figures 2,3,4,5,6)
Blinding	NA

Reporting for specific materials, systems and methods

We require information from authors about some types of materials, experimental systems and methods used in many studies. Here, indicate whether each material, system or method listed is relevant to your study. If you are not sure if a list item applies to your research, read the appropriate section before selecting a response.

Materials & experimental systems

n/a	Involved in the study	
☐	☒	Antibodies
☐	☒	Eukaryotic cell lines
☒	☐	Palaeontology
☐	☒	Animals and other organisms
☒	☐	Human research participants
☒	☐	Clinical data

Methods

n/a	Involved in the study	
☒	☐	ChIP-seq
☒	☐	Flow cytometry
☒	☐	MRI-based neuroimaging

Antibodies

Antibodies used

monoclonal anti-KDEL (Abcam; ab176333)
monoclonal anti-Tom20 (Santa Cruz Biotech., (F-10): Cat# sc-17764)
monoclonal anti-PMP70 (Abcam; Cat# ab211533)
monoclonal anti-STIM1 (BD Transduction LaboratoriesTM; Cat# 610954)
polyclonal anti-Orai (Proscience; Cat# 30-571)
monoclonal anti-β-actin (Sigma-Aldrich; Cat# A5441)
AlexaFluor secondary antibody fluorophore-conjugated (Thermo Fisher: Goat anti-Rabbit IgG AlexaFluor 405, Cat# A-31556
Goat anti-Mouse IgG AlexaFluor 405, Cat#A-31553
Donkey anti-Rabbit IgG AlexaFluor 647, Cat# A-32795)
secondary horseradish peroxidase-conjugated antibodies (Santa Cruz Biotech.; Goat anti-Rabbit IgG-HRP, Cat#sc-2004
Goat anti-Mouse IgG-HRP, Cat#sc-2005).
Mouse monoclonal anti-GFP (Santa Cruz Biotech.: Cat# sc-9996)
Polyclonal anti-β-tubulin (Cell Signaling; Cat# 2146c)

Validation

All antibodies were validated for IF and human species reactivity by the manufacturer according to their websites.

Eukaryotic cell lines

Policy information about cell lines

Cell line source(s)	HeLa ATCC
Authentication	The cell lines were not authenticated.
Mycoplasma contamination	All cell lines have been tested negative for mycoplasma contamination.
Commonly misidentified lines (See ICLAC register)	HeLa cells were used due to their easy manipulation (i.e., culture conditions, transfection efficiency and thickness for IF)

Animals and other organisms

Policy information about studies involving animals; ARRIVE guidelines recommended for reporting animal research

Laboratory animals	Danio Rerio, Sprague-Dawley rats
Wild animals	No wild animals used
Field-collected samples	All animal experiments were conducted as previously reported (Bergamin, G., Cieri, D., Vazza, G., Argenton, F. & Mostacciuolo, M. L. Zebrafish Tg(hb9:MTS-Kaede): a new in vivo tool for studying the axonal movement of mitochondria. Biochim Biophys Acta 1860, 1247-1255, doi:10.1016/j.bbagen.2016.03.007 (2016). Zebrafish embryos were obtained from spontaneous spawnings. To perform experiments, both wt and s1102t:GAL4 fish were used. All experiments were conducted on 24 hours post fertilization. (hpf) embryos. The sex of Zebrafish at 24hpf has not been determined. Adult fish were maintained and raised in 5 l tanks with freshwater at 28 °C with a 12 h light/12 h dark cycle. Sprague Dawley rats from Charles River Laboratories (Germany) were housed in the animal facility at Heidelberg University. All rats were kept in standard cages on a 12:12 h light:dark cycle with ad libitum access to food, water, and nesting material. Animals - pregnant female rats - were anesthetized with CO_2 and sacrificed using cervical dislocation. The pups were decapitated before brain removal and hippocampal primary culture preparation.
Ethics oversight	University of Padova and Italian Ministry of Education and Research for Danio Rerio for which no ethical committee is required at 24hpf, All animal procedures were carried out according to the German guidelines for the care and use of laboratory animals and in accord with the European Community Council Directive 2010/63/EU. This study was approved by the animal care and it has been registered under T-22/19.

Note that full information on the approval of the study protocol must also be provided in the manuscript.
