# Supplementary material for: An expanded palette of improved SPLICS reporters detects multiple organelle contacts in vitro and in vivo
Source: Nat Commun. 2020 Nov 27;11:6069. doi: 10.1038/s41467-020-19892-6 (PMC7699637; doi:10.1038/s41467-020-19892-6)
Supplement: Supplementary file 9 — Description of Additional Supplementary Files [file 41467_2020_19892_MOESM9_ESM.pdf]

Title: Supplementary Movie 1

Descriptions: Dynamic behaviour of ER-Mitochondria interface in Zebrafish RB Neurons, related to Figure 7b-c. s1102t:GAL4 living embryos were injected with the pT2-DsRed-UAS-SPLICSS-P2AER-MT construct, placed in low melting agarose and imaged at 24hpf. Low magnification video of 4 RB neurons expressing SPLICSS-P2AER-MT. The reconstituted fluorescence is visible both in the soma and in the axons.

Title: Supplementary Movie 2

Descriptions: Dynamic behaviour of ER-Mitochondria interface in Zebrafish RB Neurons, related to Figure 7b-c. s1102t:GAL4 living embryos were injected with the pT2-DsRed-UAS-SPLICSS-P2AER-MT construct, placed in low melting agarose and imaged at 24hpf. High magnification video of one RB neuron expressing SPLICSS-P2AER-MT, the dynamic nature of the contact sites is visible either in the soma and in the axon.

Title: Supplementary Movie 3

Descriptions: Dynamic behaviour of ER-Mitochondria interface in Zebrafish RB Neurons, related to Figure 7b-c. s1102t:GAL4 living embryos were injected with the pT2-DsRed-UAS-SPLICSS-P2AER-MT construct, placed in low melting agarose and imaged at 24hpf.High magnification inset from Movie 1 showing the movement of the contact sites in the axons of RB neurons.

Title: Supplementary Movie 4

Descriptions: Dynamic behaviour of ER-Mitochondria interface in Zebrafish RB Neurons, related to Figure 7b-c. s1102t:GAL4 living embryos were injected with the pT2-DsRed-UAS-SPLICSS-P2AER-MT construct, placed in low melting agarose and imaged at 24hpf.High magnification inset from Movie 1 showing the movement of the contact sites in the axons of RB neurons.

Title: Supplementary Movie 5

Descriptions: Dynamic behaviour of ER-PM interface in HeLa cells. Low magnification videos acquired by Leica SP5-TCS-II-RS (12 frames for second) showing the ER-PM contact sites with SPLICSS-P2AER-PM.

Title: Supplementary Movie 6

Descriptions: Dynamic behaviour of ER-PM interface in HeLa cells. High magnification videos acquired by Leica SP5-TCS-II-RS (12 frames for second) showing the ER-PM contact sites with SPLICSS-P2AER-PM.
